# Layer by Layer Engineered Lipid-Based Nanocarriers for Therapeutic Delivery and Next-Generation Design

**DOI:** 10.3390/pharmaceutics18091062

**Published:** 2026-08-26

**Authors:** Eunseok Jang, Gaeun Lee, Yoseph Seo, Hyunjun Park, Suk Min Yun, Sang Deuk Lee, Giwon Lee, Chulhwan Park, Taek Lee

**Affiliations:** 1 Department of Chemical Engineering, Kwangwoon University, 20 Kwangwoon-Ro, Nowon-Gu, Seoul 01897, Republic of Korea; bluedip28@kw.ac.kr (E.J.); garlic36@kw.ac.kr (G.L.); andy9760@kw.ac.kr (H.P.); giwonlee@kw.ac.kr (G.L.); 2 Department of Bio and Fermentation Convergence Technology, Kookmin University, Seoul 02707, Republic of Korea; akdldytpq14@kookmin.ac.kr; 3 Biological Resources Research Department, Nakdonggang National Institute of Biological Resources, Sangju-si 37242, Republic of Korea; horriwar@nnibr.re.kr (S.M.Y.); diatom83@nnibr.re.kr (S.D.L.)

**Keywords:** lipid-based nanocarrier, layer by layer system, combination therapy, hierarchical structure, bio-polymer

## Abstract

The biological fate of lipid-based nanocarriers (LBNs) is shaped at the interface. Whereas core architecture governs cargo loading, protection, and baseline release, surface architecture mediates the carrier’s initial interactions with proteins, cells, extracellular matrices, and tissue barriers, thereby influencing colloidal stability, immune recognition, targeting, biodistribution, barrier transport, and release initiation. Layer-by-layer (LbL) engineering provides a modular strategy for programming this interface through sequentially assembled coatings in which functional components are spatially separated yet mechanistically coordinated. By integrating polymers, biomolecules—including peptides and nucleic acids—and stimuli-responsive materials, LbL systems can decouple functions that are difficult to regulate independently within conventional single-layer or compositionally mixed surface architectures. This review examines recent advances in LbL-engineered LBNs (LbL-LBNs), focusing on how multilayer surface architecture reshapes physicochemical properties, cargo localization and release, biological identity, cellular interactions, and transport across physiological barriers. Particular attention is given to the multilayer interface as a dynamic biointerfacial bridge between a cargo-specific core architecture and the surrounding biological environment, including its capacity for stimuli-responsive switching in pathological microenvironments. The discussion further extends to biomimetic hybrid interfaces and establishes a framework for translating hierarchical surface architectures into reproducible, clinically tractable platforms for precision therapeutic delivery.

## 1. Introduction

The central challenge in drug delivery system (DDS) design is no longer confined to the simple encapsulation or transport of therapeutic agents. As therapeutics have expanded from small-molecule drugs to nucleic acids, proteins, antigens, and complex combination modalities, contemporary DDSs are being redefined as functional systems that must execute distinct tasks at each stage of delivery—maintaining cargo stability, negotiating biological barriers, enabling target recognition, controlling release, and modulating immunological interactions—while integrating these functions coherently at the platform level [1,2]. Accordingly, the design focus of DDSs has expanded from optimizing material composition to engineering core architectures matched to the physicochemical attributes of the cargo and, equally importantly, organizing the nanoscale surface interface at which interactions with the biological environment actually occur [3].

Among the representative DDS platforms that have evolved in response to these requirements are lipid-based nanocarriers (LBNs) [3,4]. Through combinations of phospholipids, liquid lipids, solid lipids, and ionizable lipids, LBNs can form diverse core architectures, primarily through self-assembly and emulsification [4,5,6,7]. This structural flexibility has led to a family of systems—including liposomes, lipid nanoparticles (LNPs), solid lipid nanoparticles (SLNs), and cubosomes—with distinct internal organizations tailored to the physicochemical properties of the therapeutic cargo and the intended delivery objective. Beyond the core, surface-modification strategies that coat particles with functional biomolecules, such as antibodies or polymers, have broadened LBN applications by improving interactions with biological tissues and barriers [7]. A prominent example is the LNP, which has become a central platform for mRNA vaccines and nucleic acid therapeutics. In this system, the collective interactions among phospholipids, cholesterol, polyethylene glycol (PEG)-lipids, and ionizable lipids coordinate nucleic acid complexation, colloidal stability, membrane engagement, and endosomal escape, thereby establishing LNPs as a major platform in nanomedicine [8,9,10].

Although the core architecture of an LBN can strongly influence therapeutic cargo loading, protection, and release, conventional internal structures and surface-modification approaches alone are often insufficient to independently regulate the heterogeneous events that occur at the bio-exposed interface [2,11]. Drug release control, serum-protein adsorption, target-ligand accessibility, cell-membrane interaction, and immune-cell recognition are initiated primarily at this outer interface [12,13]. When components with these distinct functions are placed on the same interface without spatial partitioning, they can interfere with one another, leading to charge neutralization, ligand masking, unpredictable protein-corona formation, heterogeneous release profiles, and reduced membrane accessibility [13,14]. Thus, an emerging design challenge for LBNs is how their outer boundary can be functionally compartmentalized and reorganized. Layer-by-layer (LbL) surface engineering has attracted attention as a strategy to address this limitation by imposing hierarchical organization on the LBN surface [15,16]. LbL assembly sequentially deposits functional layers through complementary electrostatic interactions, hydrogen bonding, hydrophobic interactions, coordination bonding, or specific molecular recognition, enabling stepwise control over the composition, thickness, charge, permeability, degradability, surface exposure, and ligand accessibility of each layer [15,17]. In this sense, LbL should be understood not merely as a surface coating, but as a strategy for structurally programming carrier function: rather than concentrating multiple functions within a single crowded composition, it separates, positions, and connects protective layers, release-regulating layers, cargo-binding layers, targeting layers, and biointerface-regulating layers at discrete layer and interface levels [16,18].

From this perspective, LbL should not be viewed as a replacement for existing LBN core architectures, but rather as a hierarchical interface strategy that redesigns the external boundary conditions of a preassembled lipid-based core. If the lipid core architecture provides the cargo-loading space, internal microenvironment, and basic colloidal framework, LbL introduces a programmable multilayer interface onto the particle surface, allowing core-level function and surface-level function to be decoupled and independently engineered [17,18]. In such an architecture, the inner layer can support core protection and cargo retention, the intermediate layer can mediate release control or stimuli-responsive switching, and the terminal layer can define the biological identity and targeting function that directly engage the biological milieu. Therefore, LbL-LBNs are more appropriately defined not as coated lipid nanoparticles, but as hierarchical delivery platforms that integrate cargo-specific lipid core architectures with programmable multilayer interfaces [18,19].

Previous reviews of LBN platforms have primarily emphasized the evolution of lipid carrier architectures or individual surface modification approaches, including PEGylation, ligand conjugation, polymer coating, and biomimetic membrane functionalization [4,20]. However, these strategies have generally been discussed as discrete functional modifications rather than as components of hierarchically organized and independently programmable surface architectures. Conversely, reviews focusing on LbL assembly and polymeric multilayer systems have mainly addressed general multilayer films, polymeric capsules, and non-lipid colloidal templates, with limited consideration of how lipid-core properties—including membrane fluidity, lipid ionization, and lipid membrane interactions—govern multilayer organization, cargo behavior, and intracellular delivery outcomes [21,22]. In addition, several reviews have examined LbL-based systems from the perspective of specific stimuli-responsive mechanisms or disease applications, but have provided limited insight into transferable design principles applicable across diverse cargo classes and therapeutic contexts [16,23]. Therefore, a comprehensive framework that connects cargo-informed design of lipid-core architectures with programmable multilayer interface organization, biological interface interactions, and translational considerations has not yet been fully established.

**Figure 1:**
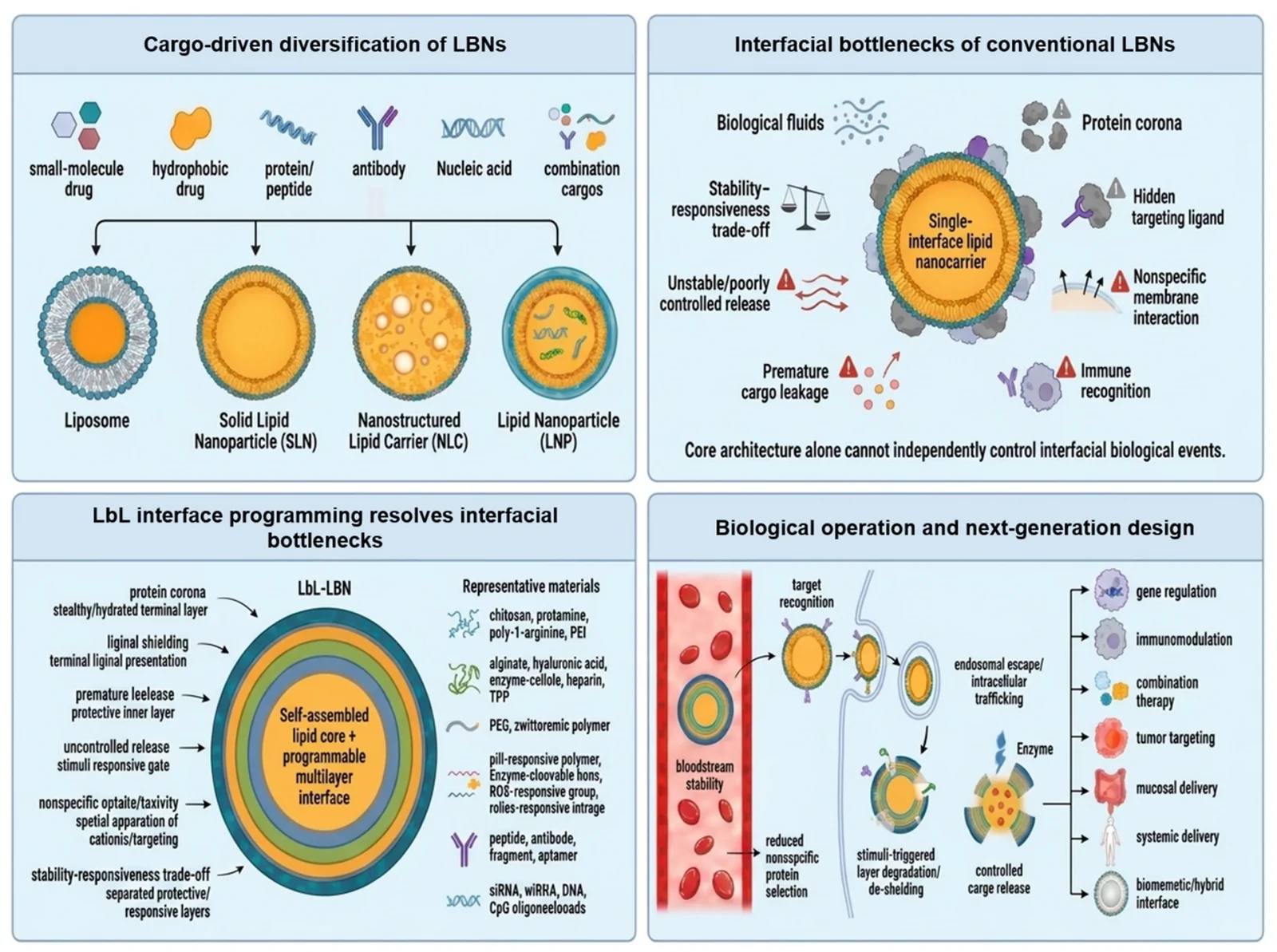
Conceptual framework of LbL-engineered LBNs. The schematic illustrates cargo-driven diversification of LBN platforms, interfacial bottlenecks of conventional lipid-based nanocarriers, and the role of programmable LbL interfaces in modulating biological stability, surface recognition, cargo release, and next-generation delivery functions.

Accordingly, this review positions LbL-LBNs as hierarchical delivery platforms that integrate cargo-specific lipid core architectures with programmable multilayer interfaces (Figure 1). The structural diversity of LBNs, conventional surface modification strategies, and the limitations of single-layer or mixed-interface designs are first summarized, followed by an examination of the fundamental principles underlying LbL interface engineering. Subsequent sections discuss LbL-induced physicochemical and structural remodeling, multilayer materials and functional interface modules, cargo localization and release mechanisms, biological interface formation, and intracellular delivery pathways. The cellular-level evaluation of LbL-LBN-based combination delivery systems and animal model-based validation of therapeutic efficacy and safety are further discussed. Finally, the extension of LbL-LBNs toward biomimetic hybrid interfaces, including exosome–liposome hybrids, exosome-like vesicles, and cell membrane-coated LBNs, together with manufacturing and regulatory considerations for clinical translation, is examined to provide future perspectives for rational LBN design.

## 2. Structural Limitations of Lipid-Based Nanocarriers and the Need for Hierarchical LbL Design

In nanomedicine, lipid-based nanocarriers (LBNs) are not only clinically validated drug delivery platforms but also a representative class of DDSs whose core architectures have progressively diversified in response to the physicochemical properties of therapeutic cargoes and formulation requirements [3]. LBNs encompass liquid lipid droplet-based lipid emulsions, lyotropic cubic phase-forming cubosomes, vesicular liposomes, matrix-based solid lipid nanoparticles (SLNs) and nanostructured lipid carriers (NLCs), as well as lipid nanoparticles (LNPs) specialized for nucleic acid complexation. The core architectures, major cargo types, and remaining formulation limitations of each LBN platform are summarized in Table 1.

### 2.1. Structural Evolution of LBNs as Drug Delivery Platforms

A decisive transition in the expansion of LBNs into compartmentalized therapeutic platforms was established through liposome research. First reported in the 1960s, liposomes evolved into representative lipid-based carriers because their structural organization, comprising an aqueous core and lipid bilayer, enables hydrophilic drugs to be accommodated within the internal aqueous compartment and hydrophobic drugs within the bilayer region [25]. Although liposomes offer the advantage of compartmentalized loading of diverse cargoes, their soft bilayer-based structure is associated with several formulation limitations, including drug leakage during storage, membrane fusion, restricted loading capacity for hydrophobic drugs, and limited control over release kinetics [26]. To address these limitations, SLNs were developed using solid lipid matrices. SLNs were designed to suppress drug diffusion and improve storage stability and sustained release; however, their high crystallinity limits drug loading capacity and can promote drug expulsion during storage as the lipid crystal lattice undergoes rearrangement [27]. To overcome these drawbacks, NLCs were introduced by combining solid and liquid lipids. By intentionally generating an imperfect lipid matrix, NLCs provide additional loading space and were designed to improve drug retention and release modulation [28].

In parallel, the expansion of nucleic acid therapeutics created a distinct set of structural requirements. Negatively charged macromolecular cargoes such as siRNA and mRNA cannot be adequately protected or delivered intracellularly by simple entrapment within lipid membranes or lipid matrices. Instead, ionizable lipid-based structures capable of condensing and stabilizing these cargoes are required [29]. Accordingly, LNPs have evolved as platforms that combine ionizable lipids, phospholipids, cholesterol, and PEG-lipids to coordinate nucleic acid complexation, colloidal stability, cell-membrane interaction, and endosomal escape [30]. In particular, the clinical success of siRNA therapeutics and mRNA vaccines has demonstrated that LNPs can serve as a central LBN platform for nucleic acid delivery [31,32].

**Figure 2:**
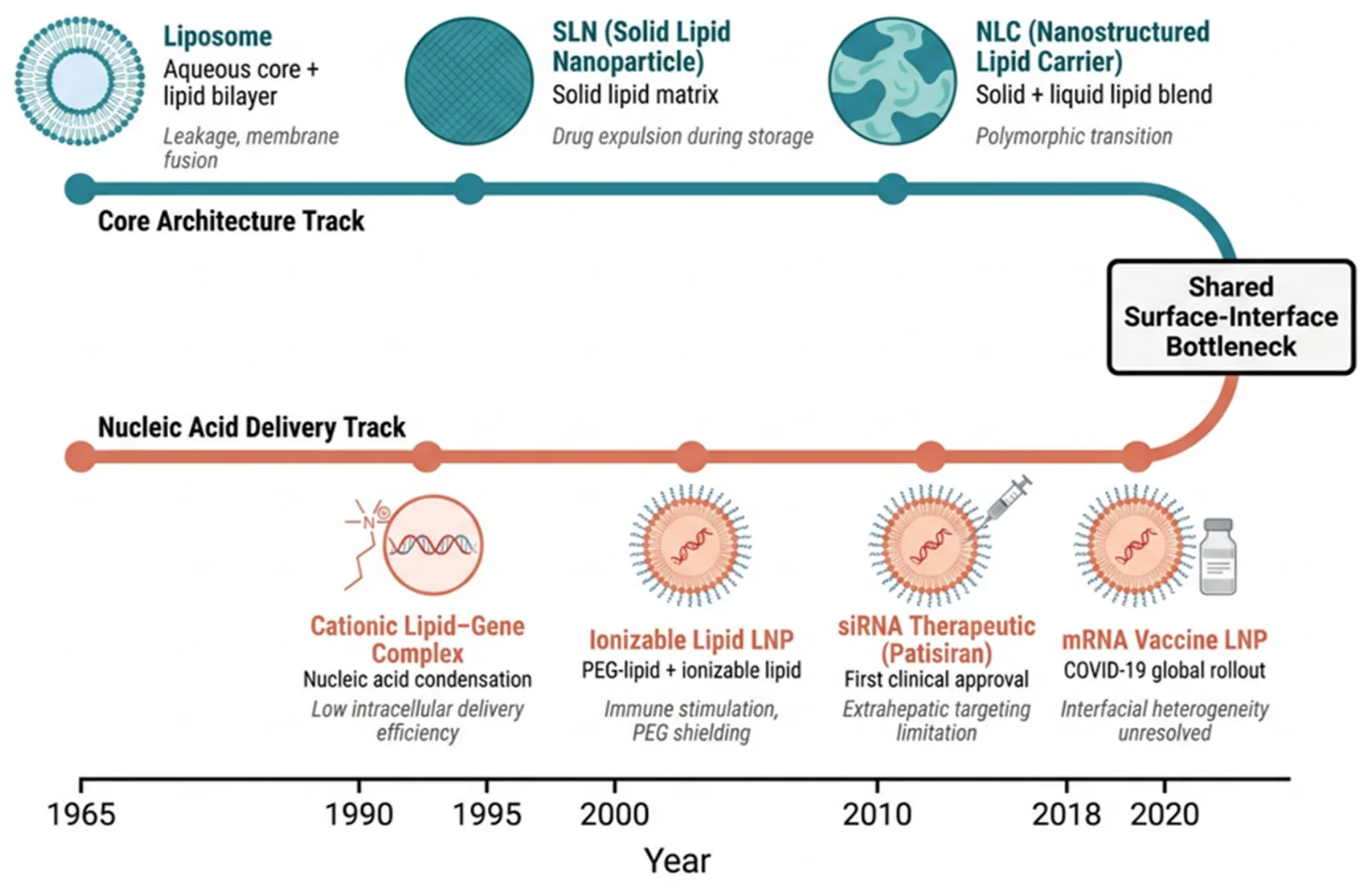
Roadmap illustrating the structural evolution of lipid-based nanocarriers.

To place these developments within the broader evolutionary landscape of LBN platforms, Figure 2 presents a roadmap highlighting the major milestones in their development. The figure illustrates two parallel trajectories: the diversification of lipid core architectures from liposomes to SLNs and NLCs, and the evolution of nucleic acid delivery systems from cationic lipid–nucleic acid complexes to ionizable LNPs, siRNA therapeutics, and mRNA vaccine platforms. Although these trajectories were driven by different cargo-related and formulation requirements, they ultimately revealed a shared limitation: the inability of conventional core architectures to independently regulate complex biological interactions at the carrier surface.

Thus, the structural evolution of LBNs is better understood not as a linear progression within a single carrier family, but as a process of architectural diversification driven by the physicochemical properties of therapeutic cargoes and formulation requirements (Table 1). Despite this diversification, however, each platform retains a common limitation at the surface interface directly exposed to the biological environment. Protein adsorption, cell-membrane contact, target recognition, immune-cell recognition, and release initiation all begin at the outer interface, yet these interfacial reactions are difficult to regulate independently using the core architecture alone [11,12]. Therefore, the subsequent development of LBNs naturally extended beyond core-architecture optimization toward surface-modification strategies that stabilize the surface interface and regulate biological interactions.

### 2.2. Surface-Modification Strategies for Self-Assembled LBNs

Once exposed to the biological environment, nanocarriers first encounter biomolecules and biological barriers through their external surface interface rather than through the internal core. Interactions at this interface directly determine blood circulation, biodistribution, cellular internalization, cargo retention, and release initiation [7,33]. Accordingly, recent efforts to enhance LBN functionality have increasingly shifted from core architecture alone toward surface-modification strategies that stabilize the interfacial boundary and regulate biological recognition and release behavior.

Early surface-modification strategies focused primarily on securing colloidal stability and prolonged circulation in biological environments. A representative example is PEGylation, in which PEG forms a hydrated layer and steric barrier on the LBN surface, thereby suppressing interparticle aggregation and fusion while reducing nonspecific serum-protein adsorption and clearance by the reticuloendothelial system (RES) [34,35]. PEG-lipids, hydrophilic surfactants, zwitterionic materials, and neutral polymer layers can likewise increase surface hydration and restrict interparticle approach, improving colloidal stability and blood persistence [35].

Surface modification subsequently expanded from simple stabilization toward the introduction of selective recognition for specific cells or tissues. Small-molecule ligands, peptides, antibodies or antibody fragments, aptamers, and transferrin have been introduced onto LBN surfaces to promote binding to cells expressing corresponding receptors [36]. Such ligand conjugation is not merely a decorative surface feature; it is a strategy intended to induce receptor-mediated uptake and thereby improve cellular selectivity and delivery efficiency. In practice, however, targeting efficiency is not determined solely by the presence or absence of a ligand. Surface density, spacer length, exposure above the PEG layer, masking by surrounding polymer layers, and the biological identity formed after serum-protein adsorption all influence targeting performance [12,36]. Thus, targeting is no longer simply a matter of attaching ligands to the particle surface, but of presenting ligands in an accessible configuration on a surface that must simultaneously retain protective functionality.

As an extension of stabilization and target-recognition control, polymer-based and biopolymer-based interfacial layers have been used to confer protection, delayed release, selective recognition, and, for certain administration routes, mucoadhesion to LBN surfaces [37]. Materials such as chitosan, alginate, hyaluronic acid (HA), and heparin can be introduced as thin interfacial layers onto preformed LBNs through electrostatic adsorption, hydrogen bonding, or chemical conjugation. Depending on their material properties, these layers can contribute to the regulation of protein adsorption, reduction in premature leakage, modulation of release rate, mucoadhesion, or receptor recognition [37,38,39,40]. However, when a single interfacial layer is expected to simultaneously function as a protective barrier, targeting layer, and release-regulating layer, it becomes difficult to independently tune layer thickness, charge, permeability, degradability, and the exposure of functional molecules [37,38]. Therefore, although polymer-based surface layers are useful for expanding LBN functionality, they still leave a structural limitation in precisely partitioning multiple functions and enabling their sequential operation.

Some surface-modification strategies have also incorporated lipid or polymer modules responsive to pH, enzymes, reactive oxygen species (ROS), or reductive environments, thereby linking release initiation to specific biological conditions. Such stimuli-responsive modifications are useful because they can reduce cargo leakage in non-target environments while inducing de-shielding, layer loosening, or cargo exposure at lesion sites or within intracellular compartments [41,42]. However, stimuli-responsive design also requires a careful balance between stability during circulation and selective activation at the target site. An overly stable surface layer may reduce responsiveness, whereas an overly sensitive surface layer can induce premature release or off-target activation [42].

Taken together, the surface-modification strategies developed for LBNs are better viewed not as a linear process of adding individual functional molecules, but as a design trajectory that has diversified to regulate stabilization, circulation, cell-membrane interaction, target recognition, and release initiation at the surface interface [7,33]. Hydrophilic shielding, charge modulation, ligand conjugation, polymer/biopolymer coating, and stimuli-responsive modification each provide clear functional advantages. Nevertheless, within single-layer or mixed-interface designs, they leave unresolved bottlenecks, including ligand masking, reduced cellular accessibility, nonspecific adsorption, toxicity, limited permeability control, and stability–responsiveness trade-offs [36,43]. As summarized in Table 2, the achievements of conventional surface modification are clear, but their limitations arise not from the absence of functional elements, but from the difficulty of separating, positioning, and operating distinct functions independently within the same interface. The focus of design therefore shifts from adding more functional components onto the surface to organizing these functions through a hierarchical interfacial architecture.

### 2.3. Limitations of Conventional Surface Modification and Application of LbL Interface Design

The limitation of conventional LBN surface modification lies not in the absence of specific functional elements, but in the functional non-orthogonality that emerges when multiple functions are placed simultaneously within a single surface interface [36]. Regulation of these functions requires different surface charge states, hydration levels, steric accessibility, permeability, and degradability, yet these variables are difficult to control independently in single-layer or mixed-interface designs [46]. This problem is therefore unlikely to be solved simply by adding more functional molecules to the surface. Rather, it indicates the need to reconstruct the surface interface itself as a functionally compartmentalized design space.

Beyond these conventional single-layer strategies, several advanced surface-engineering approaches have emerged to address specific limitations associated with functional non-orthogonality, including click chemistry-based bioorthogonal conjugation, post-insertion ligand attachment, membrane fusion approaches, polymer–lipid hybrid architectures, biomimetic membrane coating, and DNA origami-based surface engineering [47,48,49,50,51,52,53,54]. As summarized in Table 3, these strategies provide distinct advantages in improving specific interfacial functions, such as ligand precision, biomimetic recognition, or structural programmability. However, most approaches achieve functional enhancement through modification or reconstruction of a single interfacial domain rather than through the hierarchical organization of multiple functions across spatially defined surface architectures. Consequently, they retain a fundamental limitation similar to conventional surface modification strategies: multiple functionalities remain integrated within a single interface or blended surface architecture, restricting the independent regulation of protective shielding, release control, targeting, and biointerface modulation.

Building on this hierarchical design rationale, LbL-based surface engineering has recently emerged as a strategy capable of overcoming these limitations by spatially organizing distinct functional components into discrete and programmable layers [46]. This approach constructs a programmable multilayer interface by sequentially depositing functional layers onto the surface surrounding the lipid core [46,55]. The principal advantage of LbL systems extends beyond simple surface coating: protective layers, release-controlling layers, targeting layers, and biointerface-regulating layers can be hierarchically organized and precisely separated in space [39,55]. Thus, the LbL approach is regarded as a key technology that temporally and spatially decouples core formation from surface functionalization, enabling flexible switching and control of the multilayer interface [55,56].

LbL assembly can be driven by various interactions, including electrostatic interactions, hydrogen bonding, hydrophobic interactions, and covalent bonding. At present, however, electrostatic interactions remain the representative driving force for LbL assembly [57,58]. For example, when an anionic polyelectrolyte is introduced onto a cationic LBN surface, attraction between the oppositely charged surfaces is accompanied by rearrangement of hydration water and the release and replacement of counterions, providing an entropic contribution necessary for adsorbed-layer formation [57]. Once a layer is sufficiently adsorbed, the surface charge is neutralized and then reversed, and this charge reversal enables selective adsorption of the next oppositely charged layer. At the same time, excessive adsorption is restricted by surface saturation and electrostatic repulsion, giving each deposition step a degree of self-limiting behavior [57,58]. Unlike one-step self-assembly, which forms an equilibrium structure in a single event, this alternating adsorption process sequentially builds a multilayer interface on a three-dimensional colloidal surface [46,58]. Moreover, LbL can construct a common hierarchical interface on different core architectures, and once a stable colloidal core has formed, its surface can function as a scaffold for subsequent layer deposition [46,55].

From a functional perspective, the hierarchically separated layers in an LbL system can regulate complex nano–bio interactions in a stepwise manner [39,55]. For instance, the innermost interfacial layer can act as a protective network that restrains the lipid core against swelling or mechanical collapse, while primarily suppressing premature leakage of loaded small-molecule compounds or nucleic acids [55]. The intermediate layer positioned above it can serve as a stimuli-responsive release-regulating layer designed to undergo bond cleavage, swelling, or structural loosening upon exposure to specific conditions in the tumor microenvironment or intracellular endosomes, such as acidic pH, overexpressed enzymes, reactive oxygen species (ROS), or reductive environments [42,59,60]. Finally, the terminal layer, which directly contacts biological fluids, can be coated with PEG, zwitterionic polymers, or related materials to provide stealth functionality by attenuating protein-corona formation in blood and prolonging circulation time [34,35]. In addition, this outermost layer can be functionalized with chitosan, HA, peptides, or antibody fragments to confer targeting and biointerface-regulating functions, enabling precise recognition of receptors, such as CD44, expressed by cancer cells or activated macrophages [36,38]. In this modular architecture, severe steric hindrance and reduced binding efficiency that arise when targeting ligands and shielding polymers are randomly mixed on a single liposomal surface can be avoided. Moreover, the physical location and exposure sequence at which each function should operate can be finely regulated at the layer level [19,55,61,62].

## 3. Structural Characteristics of LbL-LBNs and Cellular-Level Mechanisms of Action

**Figure 3:**
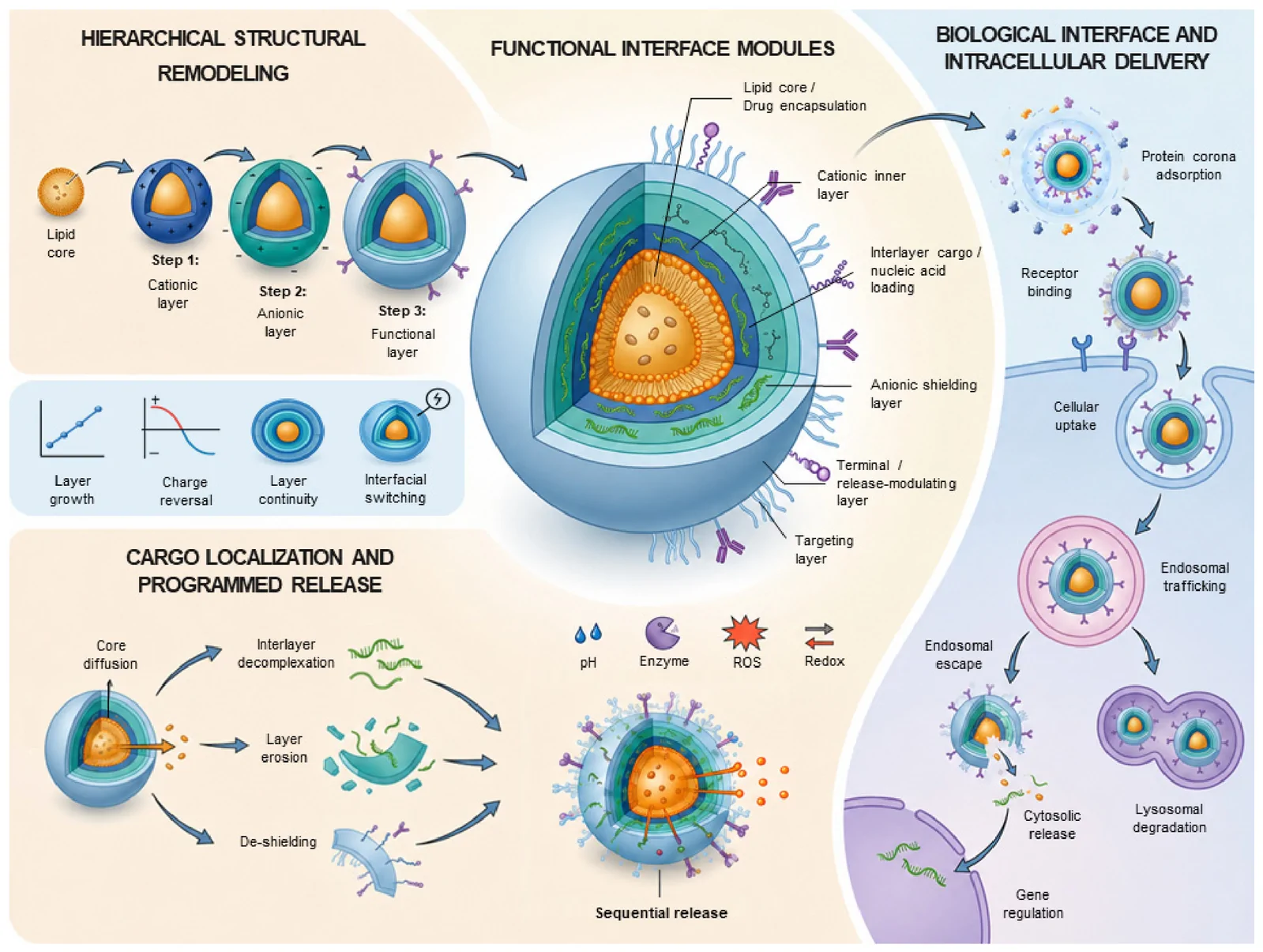
Schematic illustration of the structural characteristics and cellular mechanisms of action of LbL-LBN.

Figure 3 provides a schematic overview of the hierarchical structural organization and biological mechanisms underlying LbL-LBNs discussed throughout this section. LbL surface engineering enables hierarchical remodeling of lipid-based nanocarriers through the sequential deposition of complementary functional layers, thereby establishing a programmable multilayer interface Section 3.1. Within this architecture, individual material components are spatially organized as functional interface modules according to their designated roles Section 3.2. This layered architecture regulates cargo localization within lipid core, interlayer, and terminal layer compartments, enabling spatially and temporally controlled release through diffusion from the lipid core, interlayer decomplexation, layer erosion, and de-shielding mechanisms Section 3.3. At the biological interface, terminal layer properties govern protein-corona formation, receptor-specific interactions, and cellular uptake, thereby determining intracellular trafficking outcomes, including endosomal escape, cytosolic cargo release, and lysosomal degradation, depending on cargo characteristics and carrier design Section 3.4.

### 3.1. Physicochemical and Structural Remodeling Induced by LbL

The central concept of LbL-LBNs is not to replace the core architecture of pre-existing LBNs, but to reorganize the external boundary of a preformed lipid core into a programmable multilayer interface [55]. In this context, core–shell structures, multilayers or polyelectrolyte multilayers, functional interfacial layers, stimuli-responsive surface layers, and targeting terminal layers should not be viewed as mutually exclusive structural categories. Rather, they represent overlapping design elements that can be integrated within a single LbL-LBN surface architecture. This hierarchical remodeling is first reflected in changes in hydrodynamic size, polydispersity index (PDI), and zeta potential. Size and PDI provide information on layer growth and colloidal uniformity, whereas shifts in zeta potential or charge reversal serve as key indicators for interpreting sequential deposition and terminal layer identity [63,64]. However, these physicochemical changes alone are insufficient to establish the formation of an LbL structure. Therefore, morphology, surface composition, lipid matrix state, and structural stability should be evaluated in parallel using microscopy, Fourier-transform infrared spectroscopy (FT-IR), X-ray photoelectron spectroscopy (XPS), differential scanning calorimetry (DSC), and stability testing [65,66,67,68,69,70]. Based on this analytical framework, this section examines how LbL incorporation reorganizes the surface boundary of LBNs into a programmable multilayer interface.

#### 3.1.1. Layer Growth and Colloidal Uniformity: Size/PDI-Based Interpretation

The most immediate physicochemical changes induced by LbL assembly are alterations in hydrodynamic size and PDI. When an external layer is adsorbed onto the LBN surface, the hydrodynamic diameter generally increases as a result of the added thickness of the deposited polymer or biopolymer layer and the extension of hydrated polymer chains [64]. However, an increase in particle size alone does not necessarily indicate uniform layer growth. A stepwise increase in size accompanied by a low PDI can be interpreted as relatively uniform deposition, whereas a concomitant increase in PDI requires consideration of particle aggregation, bridging flocculation, or heterogeneous coating [63]. Thus, size/PDI-based analysis should be understood not merely as a measurement of average particle diameter, but as a primary structural readout for assessing whether colloidal uniformity is maintained during the layer deposition process.

**Figure 4:**
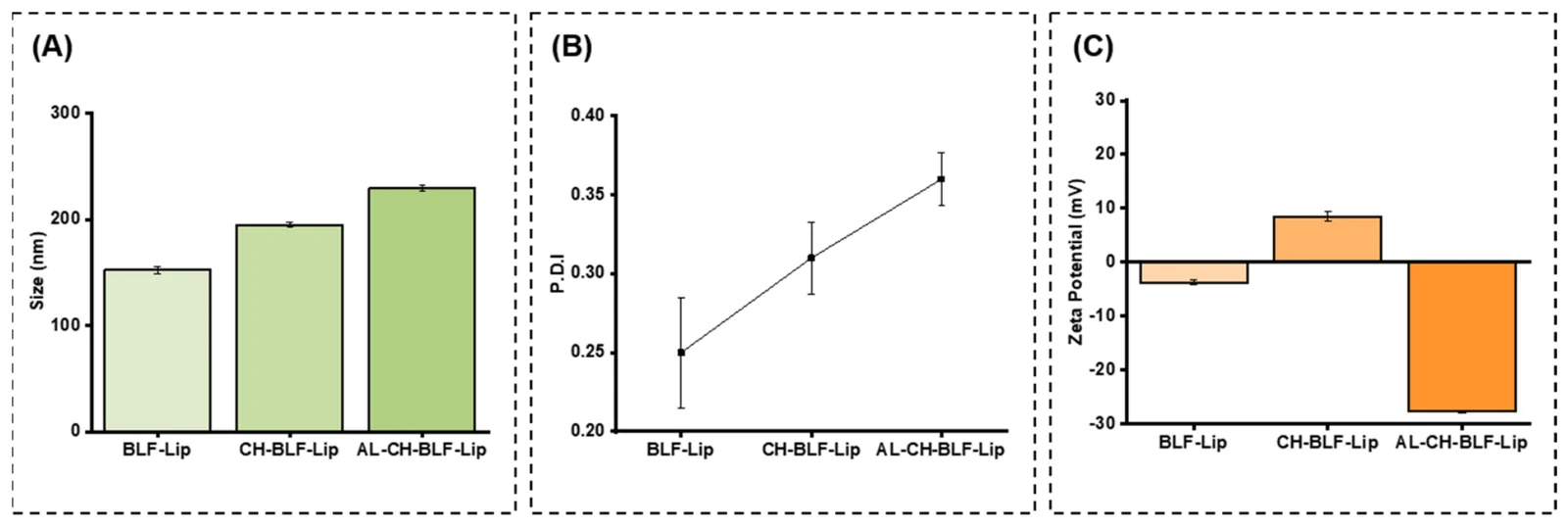
Particle size (**A**) and Polydispersity Index (**B**), Zeta Potential (**C**) of BLF−Lip, CH−BLF−Lip, AL−CH−BLF−Lip. Reproduced with permission from [71] published by MDPI, 2022.

This size/PDI-based interpretation is illustrated in studies on polymer-coated nanoliposomes. Gu et al. applied chitosan- and alginate/chitosan-based coatings to bamboo leaf flavonoid-loaded nanoliposomes (BLF-Lip) and evaluated the resulting changes in particle size and PDI after introduction of surface polymer layers [71]. Dynamic light scattering analysis showed that the average particle size of BLF-Lip was 152.13 ± 5.20 nm, which increased stepwise to 194.63 ± 4.25 nm for chitosan-coated CH-BLF-Lip and to 228.90 ± 4.89 nm for alginate/chitosan-coated AL-CH-BLF-Lip (Figure 4A). This increase suggests that the hydrated polymer shell and interfacial layer thickness increased as the cationic chitosan layer and additional alginate/chitosan coating were introduced onto the liposome surface. The PDI also increased from 0.25 ± 0.06 to 0.31 ± 0.04 and 0.36 ± 0.03, respectively (Figure 4B). Although this result indicates a modest broadening of the particle size distribution after coating, it is more appropriately interpreted as moderate size-distribution broadening associated with surface layer formation rather than abrupt aggregation or severe polydispersity.

Nevertheless, dynamic light scattering (DLS) provides an effective hydrodynamic diameter under hydrated conditions and does not directly demonstrate actual shell thickness or layer continuity. The measured size is influenced by polymer-layer hydration, ionic strength, measurement medium, and particle softness. Because DLS is also highly sensitive to scattering intensity, even a small number of aggregates can markedly alter the average size and PDI [64,72]. Thus, size increases can arise from both uniform layer growth and aggregation. For this reason, size/PDI changes represent the most intuitive first-line readout of LbL deposition, but they should be accompanied by zeta potential-based analysis of surface charge transition and microscopy-based morphology validation capable of assessing the actual particle size and surface architecture.

#### 3.1.2. Surface Charge Transition and Terminal Layer Identity: Zeta Potential and Charge Reversal

In the construction of LbL systems, zeta potential is a key indicator for interpreting the electrical identity of the exposed surface layer and the nature of the terminal layer. Zeta potential should not be interpreted as the absolute surface charge of a particle. Rather, it represents an effective electrokinetic potential that reflects exposed ionic functional groups, counterion distribution, the hydration layer, the electrical double layer, and the potential at the slipping plane [64,73]. In charge-driven LbL deposition, layers with complementary charges are adsorbed sequentially, which can lead to surface charge neutralization or reversal. For example, when a cationic polymer is adsorbed onto an anionic or weakly anionic lipid surface, the zeta potential shifts toward a positive value. Subsequent introduction of an anionic polyelectrolyte as the terminal layer can then shift the potential in the opposite direction [57,74]. Complete charge reversal, however, is not always observed in every LbL architecture. Depending on layer charge density, thickness, hydration state, polymer-chain conformation, and ionic strength, charge reversal may be only partial and should therefore be interpreted together with other characterization results.

The formulation series reported by Gu et al. clearly illustrates changes in terminal layer identity through zeta potential analysis [71]. The zeta potential of BLF-Lip, a bamboo leaf flavonoid-loaded nanoliposome, was −3.81 ± 0.79 mV, whereas chitosan-coated CH-BLF-Lip showed a shift toward positive charge, reaching +8.43 ± 1.56 mV (Figure 4C). This result suggests that the cationic chitosan layer was introduced onto the liposome surface, thereby changing the electrical identity of the terminal surface. After additional coating with an anionic alginate layer, the zeta potential of AL-CH-BLF-Lip shifted back to a negative value of −27.77 ± 0.45 mV (Figure 4C). This charge transition indicates that the charge characteristics of the outermost interface changed according to the coating sequence and supports the use of zeta potential as a useful readout for interpreting terminal layer identity.

The charge state of the terminal layer also directly influences the biointerfacial identity of LbL-LBNs. Cationic layers can strengthen interactions with negatively charged cell membranes, mucosal surfaces, and nucleic acid cargoes, but they may also increase serum-protein adsorption, complement activation, nonspecific cellular uptake, and cytotoxicity [44,45]. Conversely, anionic or hydrated neutral layers can reduce nonspecific adsorption and toxicity, but may diminish direct cell-membrane interaction or endocytic efficiency. Therefore, the target zeta potential in LbL-LBN design should not be framed simply as achieving a highly positive or strongly negative surface. Instead, it should be adjusted according to the route of administration, cargo properties, target tissue, and intended function of the terminal layer.

Several interpretive cautions are also important. Zeta potential is highly sensitive to the pH, ionic strength, buffer composition, dilution conditions, and presence of serum proteins in the measurement medium. Under PBS or physiological salt conditions, the electrical double layer can be screened, producing lower absolute values than those measured in ultrapure water. In addition, hydrated layers such as PEG, HA, or zwitterionic polymers can alter zeta potential not only by changing the actual surface charge, but also by shifting the location of the slipping plane [64,73]. Therefore, measurement conditions must be carefully considered when directly comparing zeta potential values reported across different studies, and zeta potential changes alone should not be taken as definitive evidence of LbL structure formation.

#### 3.1.3. Core–Shell Morphology and Layer Continuity: Microscopy-Based Structural Validation

If electrokinetic analysis suggests the formation of a terminal layer, microscopy-based analysis provides the next level of evidence by determining whether the deposited interface is realized as an actual spatial structure. In LbL-LBNs, the key question is not simply whether particles are present, but whether the boundary between the lipid core and the external layer is preserved, whether the coating forms a continuous shell or surface layer, and whether the LbL architecture remains intact without structural damage during the deposition process [65].

Transmission electron microscopy (TEM) can be used to examine overall particle shape, size distribution, aggregation, and electron-density contrast [75]. In systems such as LBNs, where electron-density differences may exist between the internal core and external layer, TEM can visually assess core–shell morphology, particle deformation, aggregation, and multilayer contrast. However, conventional TEM generally involves drying, staining, and vacuum conditions, which can induce deformation of soft lipid particles, shell collapse, or shrinkage of hydrated polymer layers. Therefore, cryo-TEM is more appropriate when the lipid interface and internal structure must be preserved in a near-hydrated state. By immobilizing samples through vitrification, cryo-TEM enables structural observation under conditions closer to the aqueous environment and is therefore advantageous for directly interpreting vesicular structure, lipid matrix organization, internal lamellar structure, and continuity of a multilayer shell [66].

**Figure 5:**
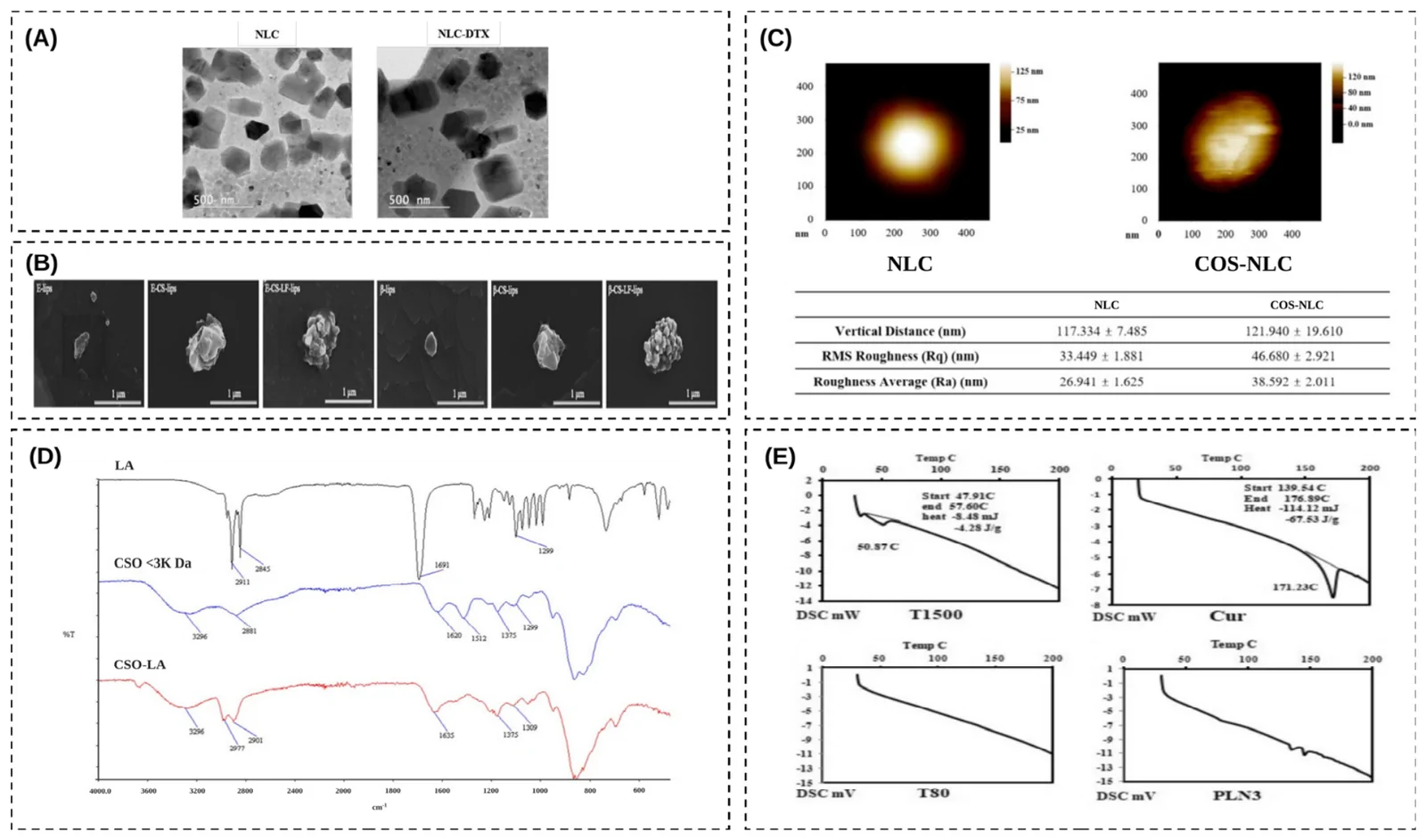
(**A**) Cryo-TEM micrographs of NLC−Blank and optimized NLC−DTX formulations at 14,500× magnification.; (**B**) SEM images of empty and β-carotene-loaded liposomes showing morphological changes based on surface modification with chitosan and lactoferrin.; (**C**) AFM analysis confirmed that both the vertical height and surface roughness of the NLCs increased after COS coating, indicating the successful formation of a polymer layer on the surface.; (**D**) The formation of CSO−LA was confirmed through a comparison of the FTIR spectra of LA, CSO, and CSO−LA.; (**E**) DSC thermograms of T1500, Cur, T80, and PLN3.; Reproduced with permission from [76,81,85] published by MDPI, 2024, 2025, 2020, and [82,90] published by Springer Nature, 2025, 2020.

The value of cryogenic transmission electron microscopy (cryo-TEM) is well illustrated in the docetaxel-loaded NLC study by Cocos et al. [76]. In that study, the nanostructures of NLC-Blank and NLC-DTX were analyzed using cryo-TEM and X-ray diffraction (XRD), and the cryo-TEM images revealed particle size, morphology, dispersion state, and internal lamellar organization. In Figure 5A, both NLC-Blank and NLC-DTX exhibited relatively uniform morphology, indicating that docetaxel encapsulation did not markedly perturb the overall particle shape or size. In particular, high-magnification cryo-TEM showed a lamellar ordered organization, suggesting that the lipid matrix of NLCs may possess an internally organized structure rather than existing as a simple amorphous particle. Although this example is not a direct case of LbL coating, it supports the use of cryo-TEM as a method capable of analyzing the internal structure and morphology of hydrated lipid nanocarriers while preserving their native-like architecture.

By contrast, scanning electron microscopy (SEM) is useful for evaluating the external surface morphology and dry-state structural integrity of particles after LbL coating. Whereas TEM and cryo-TEM are particularly suited for examining the internal structure and shell continuity of individual particles, SEM enables comparison of particle morphology, particle clustering, and fusion-like deformation over broader surface areas before and after coating [77,78]. When an external polymer or protein layer is introduced, the particle surface can shift from a smooth morphology to a rough or irregular architecture. Such coating-associated surface restructuring can be observed relatively intuitively by SEM [79]. However, SEM sample preparation often requires drying, vacuum conditions, and metal coating, all of which can shrink or deform hydrated polymer shells and soft lipid interfaces. Thus, SEM images should be interpreted not as direct representations of layer thickness in the hydrated state, but as complementary evidence of dry-state surface morphology, structural robustness, and aggregation tendency after coating [80].

Gao et al. prepared LbL-coated liposomes by sequentially introducing chitosan (CS) and lactoferrin (LF) onto the surface of β-carotene-loaded liposomes to improve the stability and bioavailability of β-carotene (βC) [81]. They compared the morphology of empty liposomes (E-lips), βC-loaded liposomes (β-lips), CS-coated liposomes, and LF-coated liposomes by SEM (Figure 5B). Before coating, E-lips and β-lips showed relatively smooth surface structures. After CS coating, an increase in particle size and a somewhat angular morphology were observed. Following the additional introduction of LF, the liposomes exhibited irregular and rough surface morphology, which can be interpreted as arising from the proteinaceous coating. These changes provide morphological evidence that the external interface was reorganized through sequential introduction of CS and LF onto the liposome surface. Nevertheless, because these results are based on dry-state SEM images, they should not be interpreted as standalone evidence confirming actual shell thickness or layer continuity under hydrated conditions. Rather, they are best viewed as supportive evidence for coating-associated surface restructuring when interpreted together with DLS, zeta potential, and compositional analyses.

Atomic force microscopy (AFM) can complement the surface topography information that TEM and SEM are less suited to provide. AFM allows analysis of height profile, vertical distance, surface roughness, local deformation, and coating uniformity, making it useful for assessing how LbL coating or polymer coating changes the nanoscale surface architecture. Lim et al. designed SR-COS-NLC by coating the surface of strontium ranelate (SR)-loaded NLCs with chitosan oligosaccharide (COS) to achieve sustained release, and used AFM to analyze surface topography changes after COS coating [82]. In Figure 5C, both NLC and COS-NLC maintained spherical morphology, but COS-NLC showed increased surface roughness. Quantitatively, the vertical distance increased from 117.334 ± 7.485 nm for NLC to 121.940 ± 19.610 nm for COS-NLC. Root mean square roughness (Rq) increased from 33.449 ± 1.881 nm to 46.680 ± 2.921 nm, and average roughness (Ra) increased from 26.941 ± 1.625 nm to 38.592 ± 2.011 nm. These results indicate that COS coating altered surface roughness and topography without substantially disrupting the overall morphology of the NLC. AFM can therefore serve as a quantitative nanoscale method for complementing the analysis of surface restructuring after coating.

Taken together, microscopy-based analyses provide morphological evidence for evaluating the hierarchical architecture of LbL-LBNs. TEM is useful for assessing overall particle morphology and contrast, cryo-TEM is advantageous for observing the internal structure of lipid particles and multilayer continuity under near-hydrated conditions, SEM allows comparison of coating-associated morphology, and AFM quantitatively complements topographical changes through surface roughness and height-profile measurements. However, in soft lipid particles, layer contrast may be weak or only partially resolved because of low electron-density differences and sample-preparation artifacts. Therefore, microscopy should be interpreted together with size/PDI, zeta potential, and the composition-based analyses discussed in the following section to more clearly define the presence and identity of external layers.

#### 3.1.4. Surface Composition and Lipid Matrix Organization: FT-IR/XPS-Based Compositional and Phase-State Analysis

FT-IR and XPS are used to verify chemical identity after LbL deposition or surface coating by examining functional-group changes and elemental composition of the external layer. In contrast, DSC is used in matrix-based LBNs such as SLNs and NLCs to evaluate changes in lipid core crystallinity, melting behavior, and drug–lipid interactions [69,70]. FT-IR is useful for confirming the introduction of coating layers and interactions among components through characteristic bands associated with amide, hydroxyl, carboxyl, phosphate, and glycosidic bonds in biopolymeric materials [83]. The appearance, shift, broadening, or intensity change in peaks can be interpreted as evidence of polymer–lipid, drug–lipid, or polymer–drug interactions [84]. For example, Noor et al. designed a dutasteride (DST)-loaded NLC surface-modified with chitosan oligomer–lauric acid (CSO-LA) to retain DST in the scalp stratum corneum while promoting follicular delivery for the treatment of androgenetic alopecia [85]. In this study, lauric acid (LA) was introduced as a hydrophobic substituent to improve CSO attachment to the NLC surface, and FT-IR analysis was used to compare the chemical identity of CSO-LA with CSO and LA. In particular, amide bond-related bands were observed near 1635 cm^−1^ and 1529 cm^−1^, arising from interactions between the carboxylic group of LA and the amine group of CSO. These bands support the successful formation of the CSO-LA conjugate (Figure 5D).

XPS is a highly surface-selective analytical method, and characteristic elemental signals can serve as chemical markers according to the composition of the external layer [86]. For example, chitosan or protein layers can be identified by nitrogen signals, sulfate-containing polyelectrolytes by sulfur signals, phospholipid- or phosphate-containing layers by phosphorus signals, and PEGylated layers by changes in the C–O/C–C ratio [86,87]. However, because XPS analyzes the surface composition within a depth of only a few nanometers under dry-state conditions, it does not directly reveal the hydrated conformation or continuity of polymer shells. Therefore, XPS provides chemical evidence supporting the identity of external layers by selectively analyzing changes in terminal surface composition after LbL deposition [67]. The study by Baek et al. provides a useful example of the value of XPS surface analysis [88]. In that study, paclitaxel- and curcumin-co-loaded SLNs were prepared for the treatment of multidrug-resistant breast cancer, and folate (FA) was conjugated to the SLN surface using stearic acid (SA) as an anchor to target folate receptors overexpressed on specific cancer cells. XPS analysis showed that nitrogen was not detected in drug-loaded SLNs without FA-SA conjugation, whereas after introduction of the FA-SA complex, nitrogen, a characteristic element of FA, was clearly observed at a concentration of 9.5%. This result suggests that surface elemental analysis by XPS can provide strong chemical evidence for successful surface modification of LBNs.

DSC can be used as a complementary method to assess the physical state of the lipid core in LbL-LBNs or coated LBNs whose structural basis is a lipid matrix, such as SLNs or NLCs. Melting peak shifts, changes in enthalpy, peak broadening, or disappearance of the drug crystalline peak before and after coating can provide clues regarding lipid matrix packing, crystallinity, drug amorphization, drug–lipid compatibility, and the possibility of storage-induced drug expulsion [89]. Abdel Fadeel et al. designed a PEGylated lipid nanocarrier (PLN3) encapsulating curcumin (Cur) in SLNs to improve the efficacy of photodynamic cancer therapy, and used DSC to evaluate the physical state of Cur and its dispersion within the lipid matrix [90]. This system was prepared using the PEGylated lipid component Tefose 1500 and Tween 80. The endothermic peaks observed in raw Cur and Tefose 1500 were no longer clearly detected in PLN3 (Figure 5E). This suggests that Cur existed in an amorphous or molecularly dispersed state within the lipid matrix rather than remaining in a crystalline form, providing thermal evidence that Cur was effectively encapsulated within the SLN core. Thus, although DSC does not directly demonstrate LbL coating itself, this case shows that DSC can serve as an important complementary indicator for assessing drug physical state and lipid core organization in matrix-based LBNs. Because DSC reflects bulk thermal responses, however, it should be interpreted together with FT-IR/XPS, microscopy, and release profiles to distinguish the contributions of the external layer and lipid core.

In summary, FT-IR, XPS, and DSC provide information at different structural levels. FT-IR interprets functional-group changes and chemical interactions among materials, XPS defines the elemental composition of the terminal surface, and DSC assesses the thermal state of the lipid core and the physical state of the drug. However, because of limitations such as FT-IR peak overlap, the dry-state surface sensitivity of XPS, and the bulk thermal nature of DSC, these analyses should not be used as standalone evidence. Instead, they should be interpreted integratively with size/PDI, zeta potential, and microscopy results. Such compositional and phase-state analyses provide the foundation for evaluating structural stability and dynamic interfacial switching, which are discussed in the following section.

#### 3.1.5. Structural Stability and Dynamic Interfacial Switching

In LbL-LBNs, stability evaluation should consider not only long-term dispersion stability, but also the extent to which the formed multilayer interface is maintained under diverse environmental conditions. In particular, it is important to determine whether the external layer remains associated with the lipid core as a hierarchical structure during storage, dilution, changes in salt concentration, pH variation, and exposure to serum proteins [91]. Even when LbL coating has been formed, the resulting surface architecture has limited functional relevance in actual delivery environments if overall stability is compromised. Therefore, structural stability should be understood as an integrated interpretive criterion for determining not only whether an LbL-LBN has formed, but also whether the hierarchical interface can be maintained under delivery-relevant conditions.

This stability is typically evaluated by comparing temporal changes in size/PDI, zeta potential, morphology, surface composition, cargo retention, and release profile [91,92]. In particular, the FT-IR/XPS/DSC-based compositional and phase-state analyses discussed above serve as a starting point for stability evaluation. For example, DSC can determine whether the drug remains in a crystalline state within the lipid matrix or exists in an amorphous or molecularly dispersed state. This initial physical state provides a basis for interpreting the likelihood of drug expulsion, leakage, or lipid matrix rearrangement during storage. Thus, stability assessment of LbL-LBNs should be designed not as the monitoring of a single numerical parameter, but as an integrated analysis of colloidal stability, interfacial integrity, cargo retention, and drug physical state.

In stimuli-responsive LbL-LBNs, however, fixed maintenance of the layers under all conditions is not necessarily desirable. Ideally, the multilayer surface architecture remains stable during circulation or in non-target environments, while de-shielding, charge transition, layer degradation, or cargo exposure is selectively induced under specific pH, enzyme, ROS, or reductive conditions [60]. In this case, zeta potential shifts, accelerated release, layer loosening, or changes in surface composition observed after stimulus treatment may be interpreted not as simple instability, but as the result of programmed interfacial switching [93]. Conversely, PDI increases, irreversible aggregation, or uncontrolled burst release that occur independently of the intended stimulus are closer to structural instability than designed switching. Therefore, structural stability and dynamic interfacial switching are important criteria for determining whether the hierarchical interface of LbL-LBNs can either be maintained under actual delivery conditions or selectively transformed in the required environment. Size/PDI, surface charge, morphology, surface composition, and lipid matrix state each provide evidence of LbL deposition and structural identity. Only when these indicators remain consistent over time and under environmental change—or transition in an intended manner—can the system be interpreted as a functional multilayer interface. This structure–property foundation leads directly to the selection of layering materials and the design of functional interface modules discussed in the next section.

### 3.2. LbL Layering Materials and Functional Interface Modules

The function of an LbL-LBN is not determined simply by the inclusion of a given material. The same material can acquire different functional meanings depending on whether it is positioned as an inner layer in direct contact with the lipid core, a cargo-associated layer, an intermediate layer responsible for release control, or a terminal layer directly exposed to the biological environment [94]. Therefore, material design in LbL-LBNs should be understood not merely as material selection, but as the design of interfacial modules in which charge, structure, degradability, and biological recognition are arranged in relation to the functional role of each layer. As discussed above, the most fundamental material module in LbL deposition is the charged layer. Cationic materials such as chitosan, protamine, poly-L-arginine, and poly-L-lysine can bind to anionic lipid surfaces, nucleic acid cargoes, or anionic polymer layers, thereby serving as interfacial bridging layers [95,96]. In contrast, hydrated anionic layer-forming materials, including alginate, hyaluronic acid, dextran sulfate, heparin, and polyphosphate, can be deposited onto cationic interfaces to attenuate surface charge and function as shielding or release-modulating layers [97,98]. However, the role of each material is determined not only by its charge polarity, but also by its position within the multilayer surface architecture.

Beyond charged layers, LbL-LBNs can incorporate protective/stealth modules, targeting modules, stimuli-responsive modules, and nucleic acid-based functional layers [99,100,101]. The roles of these modules depend less on the material class itself than on layer position and whether the module is exposed at the terminal surface. Thus, the materials summarized in Table 4 should be viewed not as a simple list of individual components, but as functional layer modules that implement circulation stability, receptor recognition, stimuli-triggered transition, and cargo-associated interface formation [94,102]. Table 4 shows how each material class operates as an interface module within LbL-LBNs, where it is typically positioned, and how it connects to cargo localization, release control, and biointerface formation. In this framework, the position and function of each material are translated into the hierarchical operating logic of LbL-LBNs. The arrangement of these material modules therefore provides the direct basis for cargo localization and release mechanisms, as discussed in the following section.

### 3.3. Analysis of Cargo Loading and Release Behavior According to LbL Structuring

In conventional LBNs, cargo loading is largely confined to the lipid core, aqueous compartment, lipid bilayer, or lipid matrix. Liposomes accommodate hydrophilic cargoes within their internal aqueous compartment and hydrophobic cargoes within the bilayer region, whereas SLNs and NLCs disperse hydrophobic drugs within the lipid matrix [107,108]. In these systems, release behavior is governed primarily by physicochemical parameters such as lipid composition, matrix crystallinity, drug–lipid affinity, and core fluidity [70,109]. With the introduction of LbL engineering, this core-centered loading paradigm expands into multilayer interface-based cargo localization. Hydrophobic drugs can still be loaded into the lipid core or lipid matrix; however, biopolymeric cargoes such as nucleic acids, peptides, and proteins, as well as antigens, adjuvants, and ionic drugs, can be positioned within the multilayer interface through complexation with charged layers, physical entrapment between layers, or presentation at the terminal layer [110,111]. Thus, cargo loading in LbL-LBNs is no longer simply a matter of increasing loading capacity. It becomes a question of designing cargo location, environmental responsiveness, and the sequence in which each function is activated.

Cargo position is closely linked to the release mechanism. Hydrophobic drugs loaded into the lipid core generally exhibit diffusion-controlled release, which is influenced by lipid-phase fluidity, crystallinity, and drug partitioning [112,113]. In contrast, nucleic acids or ionic cargoes bound within interlayers may undergo decomplexation-controlled release in response to pH, ionic strength, competitive ion exchange, or enzymatic degradation [103]. When the external polymer layer undergoes hydration, swelling, erosion, or degradation, layer-mediated diffusion or swelling/erosion-mediated release can become dominant [114]. Release from LbL-LBNs should therefore not be understood as passive diffusion from the core alone, but as a structured process governed by interfacial resistance among the core, layers, and external medium, as well as by layer permeability, binding dissociation, and stimuli-responsive degradation.

In stimuli-responsive LbL-LBNs, release can be regulated by specific biological conditions. Layers responsive to biological stimuli such as pH, redox gradients, ROS, or enzymes can reduce cargo leakage in non-target environments while promoting cargo exposure and release at lesion microenvironments or within intracellular compartments through layer loosening, de-shielding, or degradation [115,116]. In this context, the external layer functions not merely as a passive diffusion barrier, but as a switch that protects the cargo in a gate-closed state under non-target conditions and permits release in a gate-open state under target-specific conditions [117,118]. Accordingly, LbL-LBNs are more appropriately interpreted as controlled release platforms that regulate the site and timing of release according to environmental cues, rather than as carriers limited to sustained-release behavior.

**Figure 6:**
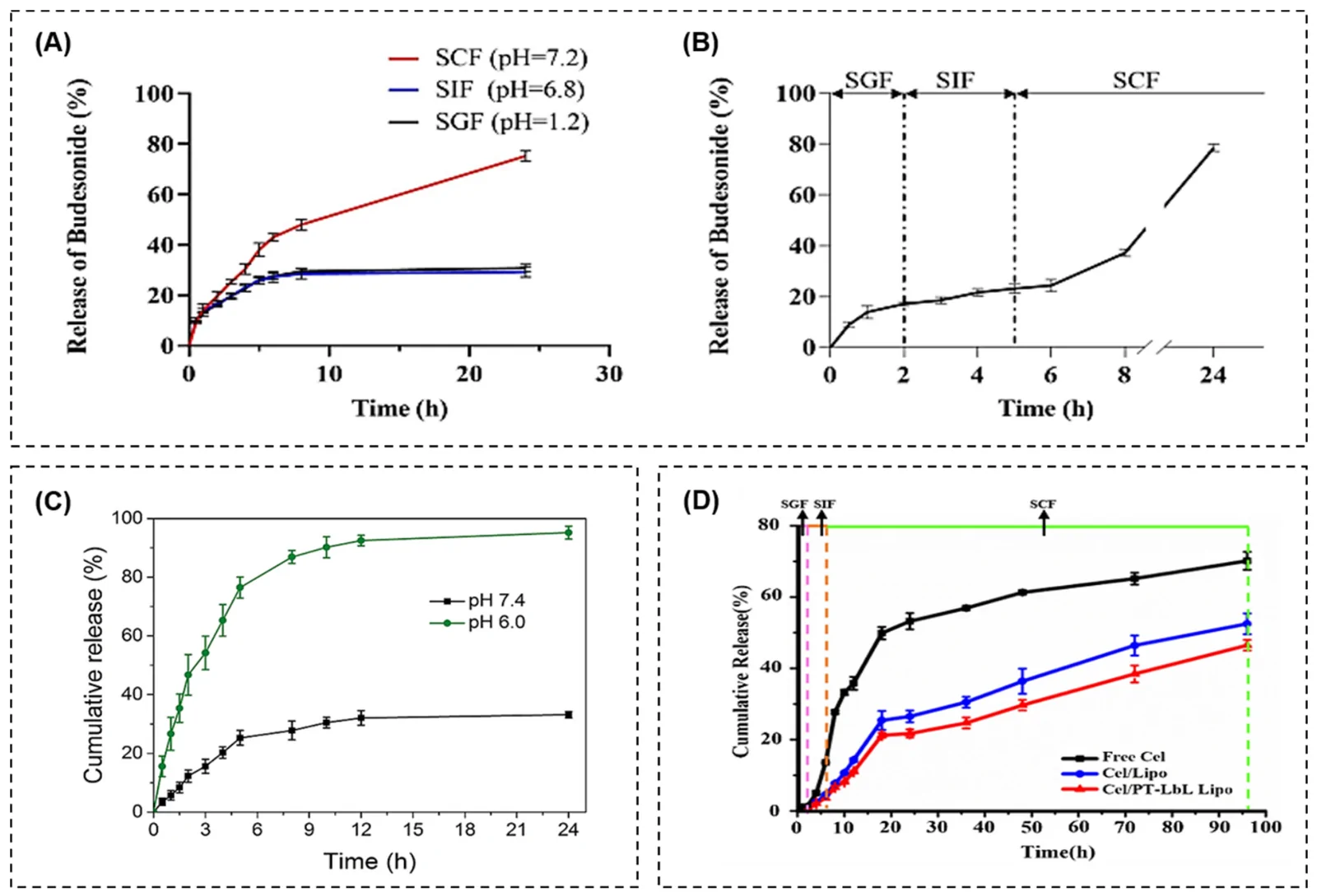
(**A**) In vitro release profiles of SLN-BUD-2L in simulated gastric, small intestinal, and colonic fluids, (**B**) Sequential drug release behavior under simulated gastrointestinal transit conditions, demonstrating enzyme-triggered colonic targeting characteristics; (**C**) pH-triggered release profile of Spe@HNPs, showing accelerated spectinomycin release in an acidic infectious microenvironment (pH 6.0) compared to physiological conditions (pH 7.4); (**D**) Release profiles of Free Cel, Cel/Liposome, and pectin/TMC-coated Cel/PT-LbL Liposome in simulated gastrointestinal fluids. Reproduced with permission from [117] published by Springer Nature, 2023, and [119] published by Taylor & Francis, 2021, and [120] published by MDPI, 2021.

This release-gating behavior is clearly illustrated by the budesonide (BUD)-loaded SLN system reported by Zhang et al. [117]. Zhang et al. encapsulated BUD, a therapeutic agent for ulcerative colitis, within SLNs and then deposited the cellulose derivative sodium cellulose sulfate and a cationic chitosan-based polyelectrolyte complex (PEC) layer through LbL assembly to produce the SLN-BUD-2L formulation. This system was designed to suppress BUD release in the stomach and small intestine, while promoting BUD release in the colonic environment through cellulase-mediated degradation of the PEC layer. In practice, SLN-BUD-2L showed low release in pH 1.2 simulated gastric fluid (SGF) and simulated intestinal fluid (SIF), whereas release was markedly increased in simulated colonic fluid (SCF) containing cellulase (Figure 6A). Under sequential release conditions mimicking gastrointestinal transit, BUD release was also promoted in the enzyme-containing environment, demonstrating that SLN-BUD-2L exhibits microbial enzyme-responsive release behavior (Figure 6B). This suggests that the external PEC layer can function as a protective layer that suppresses burst release in the upper gastrointestinal tract and as an environment-selective release gate that initiates drug release through enzymatic degradation in the colon.

Release control through a pH-responsive interface has also been demonstrated in the LbL liposome–polymer hybrid nanoparticles reported by Luo et al. [119]. To treat bacterial acute lung infection, Luo et al. encapsulated spectinomycin within a liposome core and then sequentially deposited the multivalent cationic polymer poly (β-amino ester) (PBAE) and the multivalent anionic polymer sodium alginate (NaAlg) through electrostatic interactions, generating spectinomycin-loaded layer-by-layer hybrid nanoparticles (Spe@HNPs). This system was designed to remain relatively stable at physiological plasma pH 7.4, while inducing swelling and structural changes in the polymer layers at pH 6.0, which approximates the acidic microenvironment of infected tissue. In cumulative release assays, Spe@HNPs released more spectinomycin at pH 6.0 than at pH 7.4 (Figure 6C), indicating that the LbL-based polymer interface can operate as a pH-responsive release interface that responds to the infection microenvironment.

The role of LbL coating in colon-targeted release is further supported by the celastrol (Cel)-loaded LbL liposome system reported by Xian et al. [120]. To improve the applicability of Cel, which has low aqueous solubility and limited stability, for ulcerative colitis therapy, Xian et al. prepared Cel/PT-LbL Liposome by sequentially depositing the cationic chitosan derivative trimethylated chitosan (TMC) and the an-+ionic natural polysaccharide pectin onto Cel-loaded liposomes through electrostatic LbL assembly. Pectin, used as the outermost coating material, acts as a protective layer during transit through the upper gastrointestinal tract and is hydrolyzed by pectin-degrading enzymes in the colon. As this process exposes the underlying TMC layer, TMC can adhere to mucin glycoproteins and transiently open tight junctions, thereby enhancing drug delivery efficiency. In vitro release experiments were performed for 2 h in SGF, 4 h in SIF, and 90 h in SCF. Cel/Liposome showed a more controlled release profile than free Cel, while Cel/PT-LbL Liposome exhibited an even slower release pattern (Figure 6D). These findings indicate that the pectin/TMC bilayer coating can suppress rapid Cel release and induce more sustained controlled release in the colonic environment.

Together, these examples show that release control in LbL-LBNs is not limited to simple diffusion retardation. Zhang et al.’s SLN-BUD-2L selectively increased release in the colonic environment by exploiting an enzyme gradient, Luo et al.’s Spe@HNPs regulated release through the pH difference associated with infected tissue, and Xian et al.’s Cel/PT-LbL Liposome suppressed release during gastrointestinal transit while inducing prolonged release in the colonic environment through a TMC/pectin bilayer (Figure 6A–D). In this sense, the LbL layer is not merely an external coating. It can function as an active interface that regulates cargo release in response to target-tissue enzymes, pH, mucus, or intestinal environmental conditions.

The multilayer structure is also advantageous because it can regulate the sequence of release according to cargo location. Functional molecules positioned in the terminal layer can induce early exposure, target recognition, or immune stimulation at the initial stage, whereas hydrophobic drugs loaded into the lipid core may show relatively delayed release behavior [121,122,123]. This architecture is particularly useful for combination therapies involving components with distinct temporal requirements, such as small-molecule drugs and nucleic acids, antigens and adjuvants, or membrane-interacting peptides and cytotoxic agents [95,111]. However, sequential release cannot be achieved simply by increasing the number of layers. Interlayer binding strength, cargo affinity, layer degradation rate, and core diffusion rate must be coordinated together. Excessive layering can instead cause release delay, cargo inactivation, and reduced cellular accessibility [124].

Overall, cargo loading and release in LbL-LBNs should be understood as interface-centered processes governed by how the cargo is positioned and regulated within the multilayer interface, rather than by the encapsulation capacity of the lipid core alone. By controlling cargo location within the core, interlayer region, interface, or terminal layer, LbL architecture enables cargo protection, burst-release suppression, stimuli-triggered exposure, and sequential release to be designed within a single multilayer architecture. For this release programming to translate into actual delivery efficiency, however, it is also necessary to consider what surface identity LbL-LBNs acquire between biological fluids and cell membranes, and through which pathways they enter cells. At this point, cargo arrangement and release mechanisms connect directly to biological interface formation and intracellular delivery routes, which are discussed in the next section.

### 3.4. Biological Interface Formation and Intracellular Delivery Routes

The cellular-level action of LbL-LBNs begins with the biological identity formed by the outermost layer in biological fluids. When LbL-LBNs come into contact with serum, mucus layers, or interstitial fluid, the pattern of protein corona formation can vary primarily according to surface charge, the hydration layer of the polymer, and ligand exposure [125,126]. The resulting corona reconstructs the biological identity of the LbL-LBN and affects ligand accessibility, cell-membrane adhesion, immune-cell recognition, and intracellular uptake pathways [126,127]. Therefore, cellular delivery of LbL-LBNs cannot be fully explained by the surface properties measured immediately after synthesis. It must also be interpreted in light of the actual surface state reconstructed within the biological environment.

During the initial interaction with the cell membrane, the charge of the terminal layer and the exposed functional motifs are particularly important. A cationic terminal layer can increase particle adhesion and uptake through electrostatic interactions with negatively charged cell membranes, glycosaminoglycans, and mucosal components. At the same time, however, it can also increase nonspecific protein adsorption, membrane damage, complement activation, and cytotoxicity [128,129]. Conversely, an anionic or hydrated neutral layer can reduce nonspecific adsorption, but may lower membrane accessibility and internalization efficiency [130]. Thus, surface architecture design in LbL-LBNs should not simply aim to strengthen cell adhesion. Rather, it should regulate the timing and conditions under which protective layers and cell-membrane-interacting layers are exposed.

When targeting ligands are positioned in the terminal layer, LbL-LBNs can induce selective uptake through receptor-mediated interactions beyond simple electrostatic adhesion [131,132]. However, ligand-mediated uptake is not determined solely by whether the ligand is introduced. It is strongly influenced by how exposed and accessible the ligand remains on the multilayer surface [133]. Therefore, the design of targeting layers should account for shielding by the surrounding environment and the retention of receptor-binding sites after protein corona formation, both of which can affect the targeting performance of the ligand [127,134].

**Figure 7:**
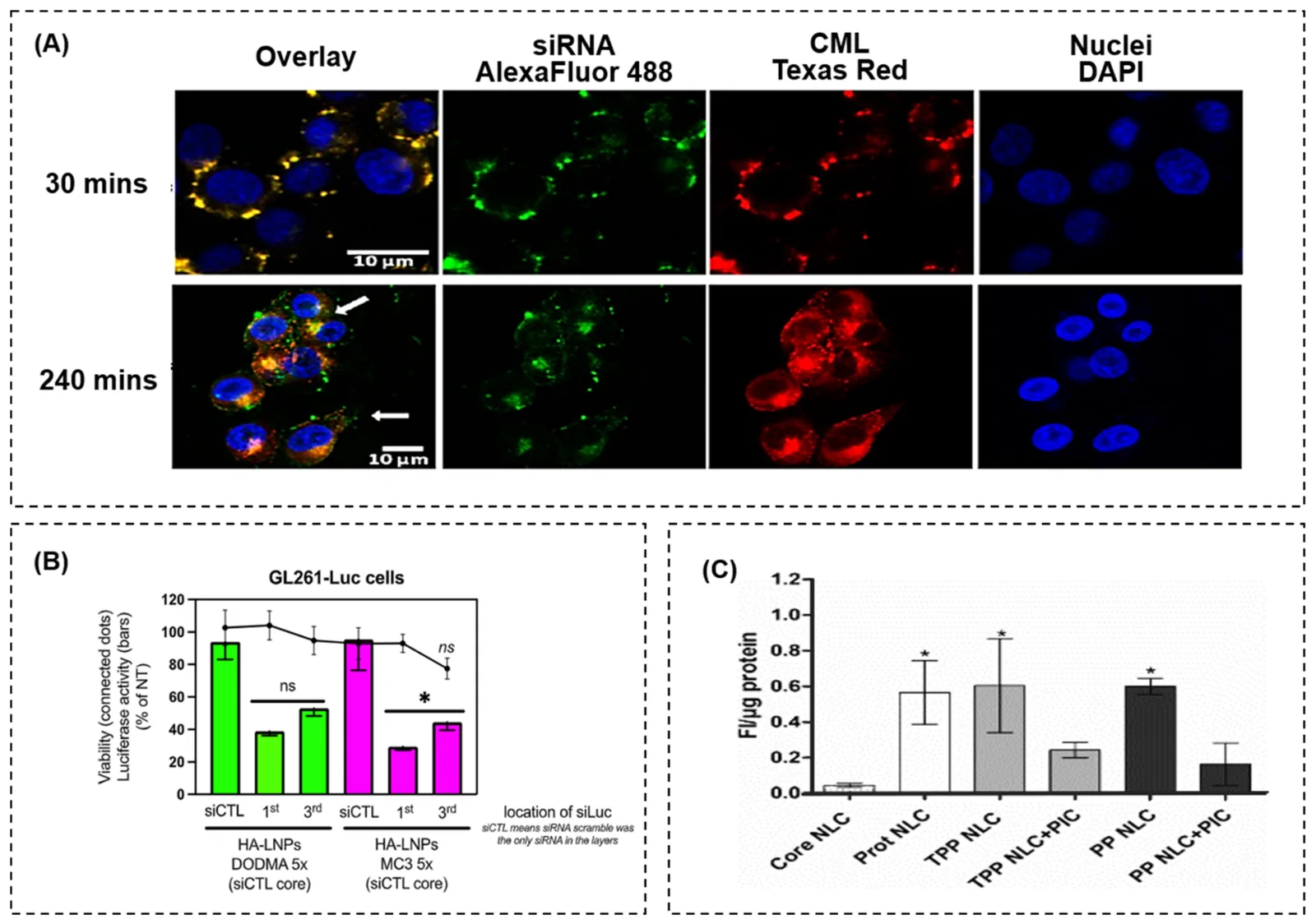
(**A**) Confocal analysis of siRNA intracellular delivery from PLA/siRNA LbL nanoparticles.; (**B**) Gene silencing activity of five-layered HA-LNPs with siLuc positioned in different LbL layers.; (**C**) ALP-dependent reversal of polyphosphate shielding significantly improves the cellular internalisation of Prot-NLCs. Reproduced with permission from [103] published by American Chemical Society, 2013, and [17] published by MDPI, 2024 and [74] published by Elsevier, 2022. * *p* < 0.05; ns, not significant.

The LbL-based liposome system reported by Deng et al. provides a representative example that visually demonstrates intracellular trafficking and cargo release [103]. In this study, an LbL system was designed to induce gene silencing in triple-negative breast cancer (TNBC) by depositing poly-L-arginine (PLA) and siRNA onto the surface of doxorubicin (DOX)-loaded liposomes, followed by introduction of HA as the outermost layer to target CD44. This produced a DOX-liposome/PLA/siRNA/PLA/HA structure. In confocal microscopy analysis, siRNA was labeled green, the liposomal core red, and the nucleus blue to track intracellular trafficking. At 30 min after treatment, the siRNA signal and liposomal core signal largely overlapped, indicating that the nanoparticles were associated with the cell membrane and that the nanoparticle structure had not yet undergone substantial disassembly. By contrast, after 240 min, the siRNA signal and core signal appeared separated, suggesting that the nucleic acid cargo within the LbL architecture dissociated from the carrier and was released intracellularly (Figure 7A).

The influence of cargo location on intracellular functionality is also demonstrated by the 5×-layered HA-LNP system reported by Gibson et al. [17]. In this study, an ionizable lipid core was loaded with siRNA, followed by construction of a five-layer siRNA/PLA/siRNA/PLA/HA architecture to improve the loading capacity and intracellular functionality of glioblastoma-related gene-silencing cargoes, including miR-181a or siRNA. In particular, alternating deposition of PLA provided cationic layers capable of interacting with nucleic acid cargoes, while HA was positioned as the outermost layer to induce CD44-mediated interaction. In Figure 7B, the gene-silencing efficiency was compared under conditions using 1,2-dioleyloxy-3-dimethylaminopropane (DODMA) or (6Z,9Z,28Z,31Z)-Heptatriaconta-6,9,28,31-tetraen-19-yl 4-(dimethylamino) butanoate (MC3) as the ionizable lipid composition, with control siRNA placed in the core and luciferase-targeting siRNA (siLuc) positioned in either the 1st or 3rd layer. The analysis showed that siLuc induced a comparable level of gene silencing not only when located in the external 1st layer, but also when positioned in the 3rd layer (Figure 7B). This result indicates that nucleic acid cargoes within LbL architecture do not need to be located exclusively in the terminal or outer layer to remain functional. Even when positioned within an interlayer, they can undergo intracellular delivery and functional release.

The ability of conditional interface exposure to promote cellular uptake is further supported by the ALP-responsive charge-reversal NLC system reported by Akkuş-Dağdeviren et al. [74]. This study addressed the limitation that a protamine layer, which can enhance cellular internalization, may be shielded by polyanionic components in vivo and thereby lose cell-penetrating efficiency. To overcome this issue, the authors designed an enzyme-responsive system by introducing a second coating layer of sodium tripolyphosphate (TPP) or sodium polyphosphate (PP), both degradable by alkaline phosphatase (ALP), onto protamine-coated NLCs. PP has longer polymer chains than TPP, whereas TPP exhibits weaker crosslinking with protamine but undergoes faster ALP-mediated charge reversal. In Figure 7C, cellular uptake was compared among core NLC, Prot-NLC, TPP-NLC, PP-NLC, and phosphatase inhibitor cocktail (PIC)-treated PIC-TPP-NLC and PIC-PP-NLC. In the presence of ALP, removal of the TPP/PP coating exposed the internal protamine layer, thereby enhancing cell-membrane interaction and cellular uptake (Figure 7C). In contrast, uptake decreased under PIC-treated conditions, supporting the involvement of enzyme-responsive de-shielding in surface charge transition and increased cellular internalization.

After cellular internalization, endosomal trafficking, lysosomal degradation, endosomal escape, and cytosolic release determine delivery efficiency [135,136]. Many LbL-LBNs undergo endocytosis and subsequently traffic from early endosomes to late endosomes and lysosomes, during which nucleic acid, protein, and peptide cargoes may be degraded or inactivated [136,137]. Therefore, for siRNA, miRNA, mRNA, and gene-editing cargoes that must act in the cytosol, endosomal escape remains a central bottleneck [138,139,140]. Conversely, when a drug is designed to be activated within lysosomes or acidic compartments, lysosomal trafficking itself can facilitate release and therapeutic activity [136].

Beyond these qualitative mechanistic descriptions, several of the studies discussed above also provide representative quantitative measurements of how LbL surface architecture affects physicochemical and cellular-level outcomes. Table 5 summarizes these values across particle characterization, intracellular trafficking timing, gene-silencing efficiency, cellular uptake, and in vitro release behavior.

These quantitative benchmarks reinforce that the functional performance of LbL-LBNs at the cellular level cannot be inferred from structural characterization alone, but must be evaluated in conjunction with dynamic biological readouts.

Taken together, the cellular-level operation of LbL-LBNs cannot be evaluated solely by the amount of uptake. The biological identity formed by the outermost layer in biological fluids, the mode of cell-membrane contact, receptor-mediated or nonspecific endocytic pathways, endosomal trafficking, and cargo release must be considered together. As shown in Figure 7A–C, LbL architecture can regulate cargo–carrier dissociation during intracellular trafficking, functional delivery of interlayer cargo, and enzyme-responsive surface activation. From this perspective, LbL surface layering is not merely a coating strategy that increases cellular uptake. It is a hierarchical delivery strategy that sequentially coordinates biological interface formation, cell-membrane recognition, intracellular trafficking, and cargo release.

### 3.5. Design Trade-Offs and Translational Challenges of LbL-LBNs

Although LbL surface engineering enables the spatial organization of distinct functionalities into separate layers, increasing layer complexity or enhancing individual surface properties does not necessarily translate into improved therapeutic performance. The impact of identical design parameters can vary substantially depending on lipid-core composition, cargo localization and mechanism of action, evaluation endpoints, and surrounding biological environments. Therefore, LbL-LBNs should not be interpreted based on isolated parameters, such as layer number or cellular uptake, but rather through a comprehensive understanding of the relationship between multilayer architecture and biological function [17,121,141]. Accordingly, comparisons among different LbL-LBN systems require consideration of terminal-layer identity, particle size/PDI characteristics, and functional outcomes rather than relying solely on individual physicochemical parameters.

An increase in layer number does not necessarily result in a proportional enhancement of therapeutic efficacy. In the aforementioned HA-decorated LNP system, Gibson et al. demonstrated that increasing the number of layers from three to five increased particle size; however, under conditions where the same amount of siLuc cargo was loaded into the core, additional layers did not further improve gene-silencing efficacy [17]. Furthermore, in five-layer MC3-LNPs, silencing activity varied depending on the localization of cargo within the multilayer structure. These findings indicate that functional delivery is not determined by layer addition itself, but rather by the integrated core–surface architecture, including cargo localization and lipid-core composition.

Similarly, cellular uptake should not be regarded as a universal indicator of delivery performance. In the PLE-terminated system reported by Barberio et al., PLE-terminated particles exhibited prolonged retention at the cell surface and functioned as a drug depot, whereas HA-terminated particles showed enhanced cellular internalization [121]. This observation highlights that increased cellular uptake does not necessarily correspond to improved functional delivery, particularly for cargos whose therapeutic activity depends on extracellular retention, receptor engagement, or sustained local activity.

Stability assessment also requires careful distinction between different biological contexts. Pires et al. reported that Mal-LbL NPs maintained their size and zeta potential for one month at 4 °C and one week at 22 °C; however, exposure to biological fluids induced LbL coating shedding and serum protein-mediated extraction of IL-12-conjugated lipids [141]. In ascites fluid, Ni LbL particles released more than 50% of IL-12 within 2 h, whereas Mal LbL particles retained approximately 70% of IL-12 even after 48 h. Therefore, storage stability and physiological persistence of the multilayer/cargo structure represent distinct concepts, and structural changes occurring in biological environments should not always be interpreted as structural instability or loss of function. Instead, such changes may represent dynamic interfacial remodeling that influences in vivo performance.

These context-dependent behaviors are closely associated with the structural and process-related limitations of conventional LbL assembly. Kashcooli et al. demonstrated that although sequential deposition of DxS and PA onto cationic liposomes induced charge inversion, cryo-TEM analysis revealed heterogeneous coatings and patch-like polyelectrolyte complexes on the particle surface [142]. In addition, resistance to salt screening varied depending on the terminal layer composition. These findings indicate that, particularly for soft liposomal LbL systems, zeta-potential reversal alone is insufficient to confirm the formation of a uniform multilayer architecture.

The influence of individual layers is also not necessarily restricted to the outermost surface. Mateos-Maroto et al. demonstrated that the charge characteristics of the terminal polyelectrolyte altered ionic cross-linking within multilayer films, thereby affecting capsule packing and mechanical rigidity [143]. Beyond structural parameters, electrostatic LbL assembly is highly sensitive to fabrication conditions. Correa et al. reported that pH, ionic strength, salt composition, and ion valency influenced polyelectrolyte adsorption and cargo loading, and that optimization of deposition and purification conditions increased nucleic acid loading by approximately eightfold [144]. These findings emphasize that solution conditions should not be considered merely experimental parameters, but rather critical process variables governing the critical quality attributes of LbL formulations.

Repeated purification steps represent another important manufacturing challenge. Conventional nanoscale LbL synthesis requires removal of excess materials after each deposition step, and Correa et al. identified intermediate purification as a major bottleneck affecting scale, yield, and production efficiency [46]. Although closed-loop diafiltration can reduce this burden, scalable translation of LbL-LBNs requires simultaneous control over not only layer number but also recovery efficiency, colloidal stability, and process consistency throughout the assembly procedure. Therefore, the central challenge of LbL strategies is not the incorporation of an increasing number of layers, but rather the establishment of a minimum functional architecture that provides the required therapeutic functions while maintaining structural definition and manufacturing feasibility [46,142,143,144].

A remaining technological challenge is the establishment of analytical frameworks capable of directly monitoring multilayer integrity and cargo behavior under physiological conditions. Measurements obtained immediately after fabrication, including size, PDI, and zeta potential, are insufficient to fully describe terminal-layer persistence or cargo–carrier association after administration. As demonstrated by Pires et al., direct evaluation of LbL coating shedding and redistribution of lipid-anchored IL-12 provides critical insight into layer-specific persistence and its relationship with biological activity [141]. Furthermore, Hadjidemetriou et al. demonstrated that complex protein corona formation on PEGylated liposomes occurs within 10 min after exposure to biological environments, with dynamic changes in individual protein abundance over time, highlighting that the biological interface of lipid nanocarriers is continuously remodeled after administration [145].

Finally, improved standardization is required to enhance reproducibility and facilitate meaningful comparison among LbL-LBN systems. Because solution conditions and purification methods can substantially influence LbL assembly and cargo loading [144], while repeated deposition–purification cycles introduce additional variability during scale-up [46], standardized evaluation criteria are essential. Future studies should establish consistent analytical frameworks that correlate layer sequence, cargo localization, layer integrity, release behavior, and batch-to-batch reproducibility with biological performance. Such approaches will be critical for advancing LbL-LBNs from highly adaptable experimental platforms toward rationally designed and clinically translatable delivery systems.

## 4. Applications and Development of LbL-LBN Systems Toward Biomimetic Hybrid Interfaces

In the biological milieu, nanocarriers do not operate simply as particles that contain and release drugs. Rather, they function as interfacial structures that continuously interact with serum proteins, immune cells, mucus layers, cell membranes, and disease-associated tissue microenvironments [146,147]. Natural biomembranes and extracellular vesicles execute complex biointerfacial functions, including immune evasion, cell-selective recognition, tissue adhesion, barrier crossing, and intercellular communication [148,149]. In the preceding sections, we discussed how the hierarchical interface of LbL-LBNs regulates not only particle properties, but also interactions with cells. The central point is that LbL-LBNs are not merely formulations in which an external layer is added onto a lipid core. They are interface-engineered delivery platforms capable of spatially separating and functionally connecting protection, release regulation, target recognition, cell-membrane interaction, and biointerface modulation within a confined nanoscale architecture. This hierarchical design can also provide a basis for linking biomimetic interfaces with combination delivery strategies [150,151]. The significance of biomimetic hybrid LBNs lies in their ability to partially reconstruct these complex biological functions at the terminal interface of nanocarriers and combine them with the cargo-loading and release-control capacity of LBNs [149,152].

In disease settings, drug resistance, inflammatory microenvironments, immune evasion, intracellular delivery barriers, and off-target organ accumulation often act simultaneously [153,154]. For this reason, a single therapeutic cargo is frequently insufficient to elicit an adequate therapeutic response, and strategies that precisely combine therapeutic agents with distinct mechanisms of action within one platform are increasingly required [154,155]. Because LbL-LBNs can subdivide internal and external structures and position different cargoes and functional modules within defined regions, they can enable division of labor among small-molecule drugs, nucleic acids, antigens, immunomodulators, and targeting modules within a single carrier [103,156]. From this perspective, this section discusses how LbL-LBN-based combination delivery systems can be evaluated. At the cellular level, functionality should first be validated in terms of cell viability, apoptosis induction, cell-cycle regulation, gene regulation, and changes in inflammatory or immune responses [155,156]. In animal models, biodistribution, cargo protection, therapeutic efficacy, lesion-site response, tissue-level effects, and systemic safety must then be interpreted in an integrated manner. Finally, we discuss chimeric LBNs, including exosome–liposome hybrids, exosome-like vesicles, and cell membrane-coated lipid nanocarriers. Although these systems do not fall within the same category as conventional polyion-based LbL-LBNs, they can be interpreted as biomimetic extensions of terminal interface programming.

### 4.1. Evaluation of Cellular Drug Responses and Combination-Therapy Effects

Combination therapy is a strategy that applies therapeutic elements with distinct mechanisms of action to overcome the limitations of monotherapy. In disease cells, drug resistance, altered gene expression, inflammatory signaling, immune evasion, and intracellular delivery barriers often act together, making it difficult for a single cargo to induce a sufficient therapeutic response [157,158]. LbL-LBNs can distinguish the lipid core, interlayer, and terminal layer, allowing different cargoes and functional interfaces to be positioned within a single carrier. This enables division of labor among small-molecule drugs, nucleic acids, antigens, immunomodulators, and anti-inflammatory agents [159]. In addition, LBNs incorporating an exosome-like surface, cell membrane-derived components, or biomimetic ligand layers can regulate cell-selective recognition, avoidance of macrophage uptake, homotypic binding, and interactions with inflammatory cells [160,161]. Therefore, in these systems, evaluation should not be limited to simple cytotoxicity. Differences in uptake between target and non-target cells, interactions with macrophages or immune cells, cytokine responses, and retention of membrane markers should also be considered. The focus of this section is not to describe biomimetic structures themselves in detail, but to clarify how such interface designs are validated as functional therapeutic responses at the cellular level.

The most basic evaluation is cell viability or cytotoxicity. Viability assays such as MTT, WST-8, CCK-8, and LDH assays can be used to compare primary therapeutic responses according to the presence or absence of a cargo or carrier [162,163,164]. However, cell viability is an endpoint that reflects the final cellular response, and a decrease in viability alone cannot explain the mechanism of combination therapy [165]. The same reduction in viability may arise from apoptosis, necrosis, cell-cycle arrest, nonspecific membrane damage, oxidative stress, or other processes [166,167]. Therefore, cell viability results should be interpreted together with functional indicators aligned with the mechanism of action and therapeutic objective of the cargo, including cell death, cell-cycle alteration, oxidative stress, inflammatory responses, and gene-expression regulation. In anticancer drug-based LbL-LBNs or related co-delivery LBNs, apoptosis, caspase activation, mitochondrial dysfunction, cell-cycle arrest, and changes in proliferation markers serve as important readouts. By contrast, for anti-inflammatory or immunomodulatory systems, it is necessary to determine whether inflammatory markers such as NO, cytokines, iNOS, COX-2, and NF-κB-related markers are regulated while minimizing cytotoxicity. For systems containing nucleic acid cargoes, changes in target mRNA or protein expression are more important than uptake alone. In other words, the fact that nucleic acids enter cells must be distinguished from the actual occurrence of gene silencing, miRNA replacement, or immune activation. These evaluation parameters and interpretive criteria are summarized in Table 6.

Comparison-group design is particularly important when claiming a combination-therapy effect. A formulation carrying two cargoes does not prove synergy simply because it induces a stronger cellular response than a single-cargo formulation. The therapeutic effect should be compared not only according to the drug combination, but also according to the presence or absence of LbL engineering. Whenever possible, the combination effect should be quantified using a dose–response matrix in which the concentrations of the two cargoes are systematically varied. The combination index distinguishes synergy from an additive effect based on the dose reduction required under combination treatment, whereas Bliss independence compares the observed combination effect with the expected effect assuming independent action of the two cargoes [168,169]. Combination effects in current drug combinations can be quantitatively evaluated using reference or null models, including Highest Single Agent (HSA), Bliss independence, Loewe additivity, and Zero Interaction Potency (ZIP) models [169,170,171,172,173]. The HSA model evaluates whether the combination response exceeds the effect of the most active single agent [169,170], whereas the Bliss independence model calculates the expected combination effect based on the assumption that two agents act independently [171]. The Loewe additivity model assesses dose additivity by comparing the doses of individual agents required to achieve an equivalent therapeutic effect [172]. In contrast, the ZIP model defines zero interaction as a condition in which combination treatment does not alter the dose–response potency relative to individual agents [173]. Because these models are based on different assumptions regarding single-agent behavior and non-interaction states, distinct synergy interpretations may be obtained from the same combination dataset. Therefore, combination effects should not be interpreted solely based on a single synergy score, but rather by considering the underlying assumptions and limitations of each reference model [170].

However, these reference models primarily estimate expected combination responses from single-agent and combination dose–response relationships and do not explicitly incorporate carrier-specific parameters, such as cargo localization, multilayer architecture, or temporally controlled release behavior [169,170,173]. This limitation should be considered when evaluating LbL-LBN-based dual-cargo delivery systems. In LbL-engineered liposomal platforms, different therapeutic cargos can be spatially separated between the lipid core and polyelectrolyte layers, resulting in cargo-specific release kinetics and distinct exposure profiles [19]. For example, a doxorubicin-loaded liposome coated with a siRNA-containing LbL film exhibited independent release behaviors of the two therapeutic components, demonstrating that multilayer organization can regulate cargo availability beyond simple co-encapsulation [103]. Furthermore, recent studies have shown that LbL architectures may undergo dynamic remodeling under physiological conditions, including polymer coating shedding and time-dependent extraction or release of IL-12-conjugated lipids from the liposomal core in the presence of serum proteins [141]. Therefore, although dose–response-based synergy models provide valuable quantitative frameworks for comparing pharmacological interactions, they cannot independently explain how LbL architecture contributes to cargo-specific release, temporal exposure, or in vivo delivery behavior. Future evaluation strategies should integrate conventional reference models with measurements of multilayer architecture, cargo localization, layer-dependent release profiles, and temporal exposure patterns to distinguish pharmacological synergy from delivery-system-mediated functional enhancement [19,103,141,170]. When such quantitative analyses are not performed or remain insufficient to establish a true interaction effect, restrained expressions such as enhanced combination effect or improved co-delivery response are more appropriate than claiming synergistic effects.

Barberio et al. designed PLE-IL-12-NPs by attaching 6 × His-tagged interleukin-12 (IL-12) to the surface of liposomes and then sequentially depositing poly-L-arginine (PLR) and poly-L-glutamic acid (PLE), with the aim of reducing the systemic toxicity of IL-12 while preserving its immune activity within the tumor microenvironment [121]. PLR was used as a cationic intermediate layer to support LbL deposition and stability, whereas the outermost PLE layer was designed to allow the carrier to remain on the tumor-cell surface for a longer period rather than being rapidly internalized, thereby providing sufficient time for IL-12 to interact with membrane receptors. In vitro, the functional activity of IL-12 was confirmed by IFN-γ production in splenocyte and tumor cell–splenocyte co-culture systems. In vivo, tumor growth and survival were evaluated in MC38 and HM-1 tumor models. PLE-IL-12-NPs maintained antitumor efficacy comparable to that of free IL-12 while reducing body-weight loss and serum IL-12/IFN-γ elevation, showing that cytokine-based LbL-LBNs can simultaneously regulate immune activity and safety.

**Figure 8:**
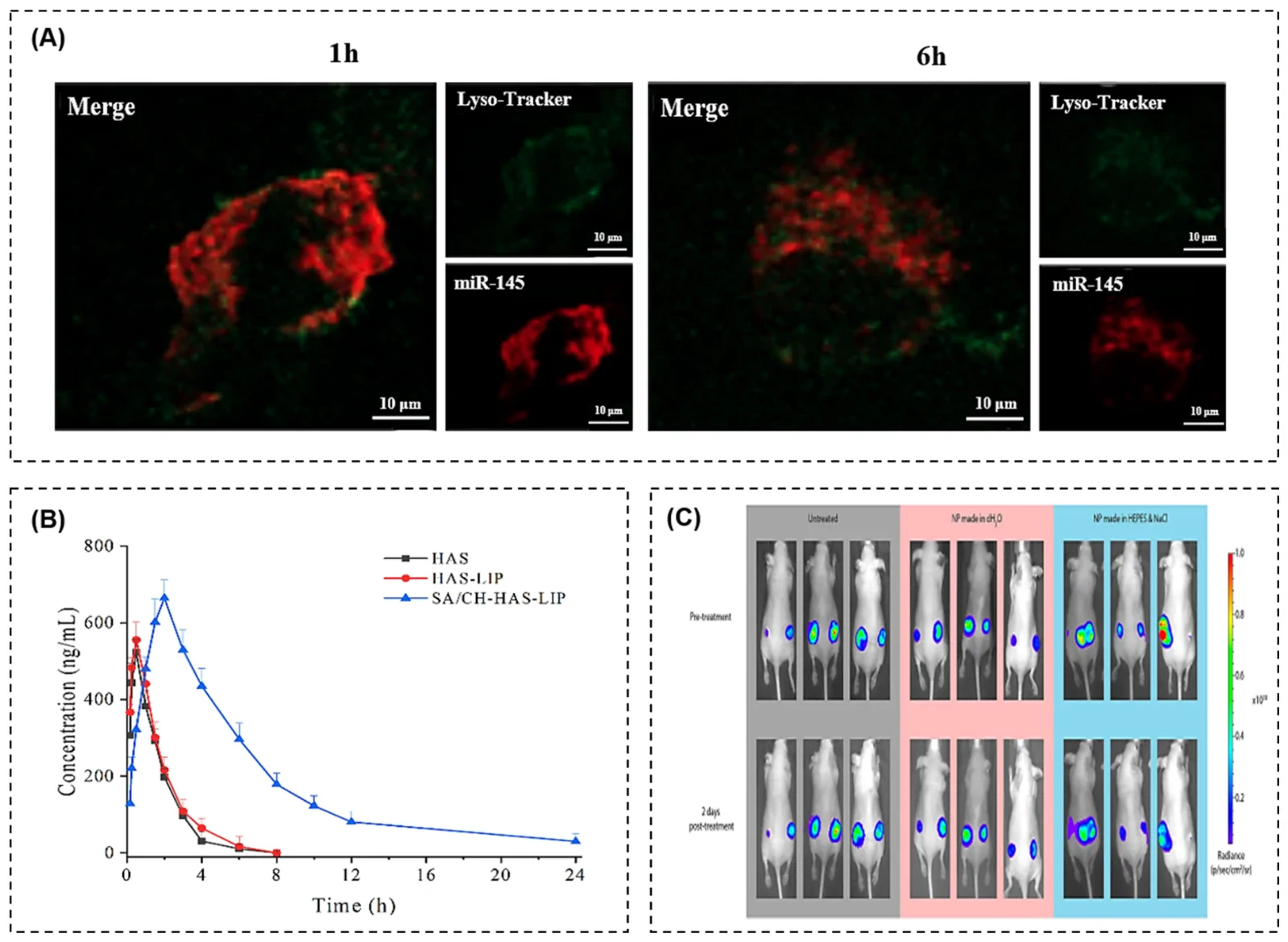
(**A**) Analysis of the lysosomal escape process of LA-CMGL by observing the localization of miR-145 (red) and Lyso-Tracker (green) at 1 and 6 h using confocal laser scanning microscopy (CLSM).; (**B**) Changes in plasma drug concentrations following the administration of HAS, HAS-LIP, and SA/CH-HAS-LIP to rats.; (**C**) In vivo luciferase silencing by HEPES/NaCl-optimized siLNA-loaded LbL liposomes in OVCAR8 tumors. Reproduced with permission from [174] published by Springer Nature, 2024, and [197] published by MDPI, 2023 and [144] published by American Chemical Society, 2019.

The LA-CMGL system reported by Rong et al. provides a related example of how surface targeting and multi-cargo delivery in an LBN-based combination system can be connected to cellular functional readouts [174]. To mitigate the toxicity, adverse effects, and resistance associated with camptothecin (CPT), a drug used for hepatocellular carcinoma therapy, this study designed an LBN-based LA-CMGL formulation functionalized with lactobionic acid (LA) on the surface. The formulation was constructed to contain CPT together with miR-145, which regulates tumor-associated gene expression, and Gd-DOTA as a magnetic resonance imaging (MRI) contrast agent. miR-145 suppresses SENP1, which stabilizes the HK2–VDAC1 interaction, thereby weakening the metabolic and structural defense machinery of cancer cells. Once this defense axis is disrupted, CPT can deliver a stronger cell-death signal, amplifying the combination-therapy response and inducing an effective anticancer effect. Although this case is not a typical LbL-deposited architecture, it represents an LBN-based application demonstrating that surface-ligand regulation and combination cargo delivery can improve therapeutic efficacy. Confocal laser scanning microscopy (CLSM) was used to monitor the lysosomal escape of LA-CMGL at 1 and 6 h. At 1 h after administration, LysoTracker signal (green) and miR-145 signal (red) overlapped, indicating that the nanoparticles had entered the cells but remained entrapped within lysosomes. By contrast, at 6 h, the overlapped signals began to separate, confirming that the carrier induced drug release from lysosomes into the cytosol. These results demonstrate that the carrier enabled effective intracellular delivery by facilitating lysosomal escape and subsequent cargo release into the cytosol (Figure 8A).

Taken together, the combination-therapy potential of LbL-LBNs at the cellular level cannot be evaluated using a single endpoint such as cell viability. The key question is not simply whether different cargoes are loaded into the same particle, but whether each cargo is protected at the intended location, released under the appropriate conditions, and translated into a functional therapeutic response at the cellular level. Therefore, the focus of this section should not be to repeat the structural characteristics or intracellular uptake pathways of LbL-LBNs, but to evaluate whether hierarchical interface design leads to a functional therapeutic response in combination delivery.

**Table 6:** In vitro functional evaluation framework for LbL-LBN-based combination delivery systems.

Evaluation Category	Representative Methods	Measured Parameters/Readouts	Evaluation Purpose/Interpretation	Ref.
Cell viability /cytotoxicity	MTT, WST-8, CCK-8 assay	Metabolic activity-based cell viability	Comparison of differences in first-line treatment response among free cargo, single-cargo LBN, dual-cargo LBN, and LbL-coated LBN	[162,163,164]
	LDH release assay	Membrane damage, cytotoxicity	Distinguish whether the reduction in viability is due to metabolic suppression or cytotoxicity resulting from membrane disruption	[162,165]
Cell death	Annexin V/PI staining	Early apoptosis, late apoptosis, necrosis	Determine whether the decrease in cell viability is linked to apoptosis or necrosis	[166]
	Caspase-3/7 or caspase-9 assay	Caspase activation	Determine whether the apoptotic signaling pathway is activated	[167]
	TUNEL assay	DNA fragmentation, apoptotic nuclei	Detection of apoptosis-associated DNA damage at the tissue or cellular level	[175]
Cell cycle /proliferation	PI staining-based cell cycle analysis	G0/G1, S, G2/M phase distribution	Determine whether cell cycle arrest or proliferation suppression has occurred	[176]
	Ki-67 staining, EdU/BrdU incorporation assay	Proliferation marker, DNA synthesis	Check for inhibition of cell proliferation	[177,178]
	Western blot	Cell-cycle-related proteins such as cyclin, CDK, p21, p27, p53, etc.	Supplementing the molecular basis for cell-cycle arrest	[179]
Oxidative stress/mitochondrial damage	DCFH-DA assay	Intracellular ROS	Assessment of increased oxidative stress or antioxidant effects	[180]
	MitoSOX staining	Mitochondrial ROS	Determination of mitochondria-associated oxidative stress	[181]
	JC-1 assay	Mitochondrial membrane potential	Determine the link between mitochondrial dysfunction and apoptosis	[182]
	GSH assay	Intracellular glutathione level	Assessment of redox balance or recovery from oxidative damage	[183]
Inflammatory response	Griess assay	Nitrite/NO production	Assessment of the inhibition of inflammatory NO production; the Griess assay results are interpreted solely based on the NO/nitrite readout	[184]
	ELISA	Secreted cytokines, e.g., TNF-α, IL-6, IL-1β, IL-10	Assessment of changes in the secretion of pro-inflammatory or anti-inflammatory cytokines in the culture medium	[185]
	qPCR/RT-qPCR	mRNA levels of inflammatory genes, e.g., iNOS, COX-2, TNF-α, IL-6	Determine whether the expression of inflammation-related genes is regulated	[186,187]
	Western blot	iNOS, COX-2, NF-κB p65, p-p65, IκBα, MAPK-related proteins	Assessment of changes in protein levels within inflammatory signaling pathways	[179]
Nucleic acid cargo function	qPCR/RT-qPCR	Target mRNA level, miRNA level	Verify whether the siRNA/miRNA/mRNA cargo functioned effectively within the cells	[186,187]
	Western blot	Target protein expression	Verify whether the changes in mRNA led to actual protein regulation	[179]
	Reporter assay	Luciferase or fluorescence reporter activity	Verification of functional readouts for nucleic acid cargo, such as gene silencing, miRNA replacement, and immune activation	[188]
Biomimetic/biointerface response	Flow cytometry, confocal microscopy	Target vs. non-target cell uptake, macrophage uptake, homotypic binding	To determine whether the biomimetic interface or terminal ligand layer modulates cell selectivity, immune cell evasion, and cell membrane interactions	[160,189]
	Western blot, immunostaining	Membrane marker retention, e.g., CD47, CD44, integrin, tetraspanins	Verification of the maintenance of biological identity in cell membrane-derived or exosome-like interfaces	[160,189]
	ELISA/cytokine assay	Cytokine response after immune cell exposure	Evaluation of whether biomimetic interfaces induce or mitigate immune responses	[185,189]
Combination effect	Dose–response curve	IC50, effective concentration, dose reduction	To determine whether co-delivery improves therapeutic response compared to single treatment	[168,169]
	Combination index analysis	CI value, dose reduction index	Distinguishing between additive and synergistic effects using methods such as the Chou-Talalay method	[168]
	Bliss/Loewe/HSA model	Expected vs. observed response, synergy score	Quantitatively evaluate whether the combination effect arises from a simple increase in concentration or from complementary mechanisms	[169]

### 4.2. In Vivo Biodistribution, Stability, and Therapeutic Efficacy in Animal Models

In vitro systems can directly assess responses such as cellular uptake, changes in cell viability, apoptosis, cell-cycle regulation, and gene expression. However, they do not fully recapitulate the actual blood environment, including plasma protein adsorption and immune-related interactions [190,191]. Therefore, in vivo evaluation of LbL-LBNs in animal models is required to verify whether, after administration, the carrier reaches the target tissue and whether each surface layer maintains and expresses its function through interactions with cells and biological barriers.

Among these parameters, biodistribution and in vivo fate should be evaluated first. Fluorescent labeling, radiolabeling, MRI contrast agents, and ex vivo organ imaging can be used to assess organ distribution and target-tissue accumulation over time after ad-ministration [192,193]. However, a distribution signal reflects the movement of the carrier or label and does not directly demonstrate that each therapeutic cargo has functionally reached the same site. In particular, because the in vivo stability of the core cargo, interlayer cargo, and terminal layer in LbL-LBNs may differ, a single labeling signal is insufficient to establish functional delivery of the entire formulation. In systems containing nucleic acid cargoes, protection from nuclease-mediated degradation is especially important. For hydrophobic drugs, suppressing premature release and maintaining a sufficient therapeutic concentration at the target tissue are required [194,195,196]. In addition, LbL structures may undergo external-layer desorption, serum-protein ex-change, terminal-layer masking, or cargo leakage. Thus, serum stability, circulation time, cargo retention, release profile, and changes in zeta potential or size should be evaluated together [145]. In vivo stability, in this sense, is not merely a question of how long the particle circulates, but whether the hierarchically positioned cargoes and surface functions are preserved in the biological environment.

Tan et al. designed SA/CH-HAS-LIP to overcome the limitations of oral delivery for hydroxy-α-sanshool (HAS), whose activity decreases in the gastrointestinal environment. In this system, HAS was encapsulated in liposomes, and the surface was coated with a sodium alginate/chitosan complex composed of sodium alginate, which improves drug stability, and chitosan, which enhances permeability and oral bioavailability [197]. Figure 8B shows the blood concentration–time profiles after administration of free HAS, HAS-liposome, and sodium alginate/chitosan-HAS-liposome to rats. Free HAS and HAS-liposome rapidly increased in blood concentration after administration and were nearly eliminated from the body after 8 h. By contrast, sodium alginate/chitosan-HAS-liposome showed a higher maximum blood concentration and a more gradual decline. This suggests that the SA/CH coating layer acted as a protective barrier in the gastrointestinal environment and improved the stability and systemic residence of HAS. As a result, the oral bioavailability of sodium alginate/chitosan-HAS-liposome increased 4.6-fold compared with free HAS and 4.2-fold compared with HAS-liposome. This case demonstrates that LbL like coating can alter in vivo exposure, circulation profile, and oral bioavailability beyond simple surface modification.

Delivery efficiency and preservation of cargo function are even more critical evaluation criteria in nucleic acid-based LbL systems. Correa et al. designed an LbL liposome by sequentially depositing poly-L-arginine (PLR), a siRNA analogue (siLNA), PLR, and pro-pargyl-modified poly-L-aspartic acid (pPLD) onto an anionic liposomal core [144]. In this system, PLR functioned as a cationic polypeptide layer supporting charge conversion and nucleic acid delivery, siLNA served as the gene-silencing cargo, and pPLD acted as the final anionic capping layer. Figure 8C shows the evaluation of in vivo gene silencing in luciferase-expressing OVCAR8 xenograft model using in vivo imaging system-based bioluminescence imaging. The optimized formulation assembled under HEPES/NaCl conditions and purified with DI water induced a more pronounced reduction in tumor luciferase signal at day 2 than nanoparticles prepared under DI water conditions. This result indicates that optimization of assembly conditions can improve siLNA loading and LbL structural stability, ultimately leading to functional nucleic acid delivery in vivo.

Nevertheless, superior therapeutic efficacy of dual-cargo or nucleic acid-loaded lipid nanocarriers does not automatically prove that the effect results from hierarchical structure or combination-delivery design. To rigorously interpret the contribution of combination therapy or LbL architecture, stepwise comparisons are required among free cargo mixtures, single-cargo formulations, dual-cargo formulations, uncoated LBNs, layer-engineered LBNs, and LbL-LBNs. These comparison groups are necessary to distinguish whether the increased therapeutic effect arises from a simple increase in administered dose, parallel action of two cargoes, carrier-mediated stabilization, or LbL-mediated cargo protection and release regulation [198]. In particular, to claim the advantage of LbL-LBNs, it is necessary to experimentally separate which stage is affected by the multilayer interface: cargo stability, target-tissue delivery, release timing, or terminal biointerface formation.

Finally, safety evaluation is not an auxiliary experiment independent of therapeutic efficacy. It is an essential axis for interpreting combination delivery platforms. Because LbL-LBNs may contain cationic layers, nucleic acid cargoes, immune-active components, PEG-lipids, targeting ligands, or biologically derived membrane components, biological safety must be evaluated from multiple perspectives beyond cytotoxicity [199,200,201]. In combination therapy systems, it is particularly important to distinguish whether enhanced therapeutic efficacy reflects an improved therapeutic index or simply increased drug exposure or toxicity. Body-weight changes and clinical symptom monitoring can serve as primary indicators of systemic toxicity, whereas hematological parameters are required to evaluate blood toxicity such as immune-cell alterations, anemia, and thrombocytopenia [202]. Alanine aminotransferase (ALT) and aspartate aminotransferase (AST) reflect potential liver injury, whereas blood urea nitrogen (BUN) and creatinine reflect changes in renal function. Hematoxylin and eosin (H&E) staining of major organs can be used to assess tissue-level inflammation, necrosis, edema, or structural damage [202,203]. When inflammatory cytokine profiles such as TNF-α, IL-6, and IL-1β are analyzed together, systemic inflammatory responses or immunotoxicity induced by LbL-LBNs or combi-nation cargoes can be interpreted more comprehensively [204].

### 4.3. Programmable Combination Platforms Extended to Biomimetic Hybrid Interfaces

Recently, the concept of hierarchical interface design has expanded beyond synthetic polymers or polyion-based layering toward biomimetic hybrid interfaces. Exosome-LBN hybrids and cell membrane-coated LBNs represent major strategies that combine the bio-logical identity of biomembranes with the formulation flexibility of LBNs [53,205]. Although these systems should not be regarded as belonging to the same category as conventional LbL-LBNs, they show structural continuity with the hierarchical surface architecture concept of LbL-LBNs because they reconstruct the terminal interface by introducing biomembrane-derived interfaces onto lipid-based cores or vesicular structures [53,151]. In particular, exosome- or cell membrane-derived interfaces can confer biological functions such as target recognition, immune evasion, cellular affinity, and in vivo stability onto the carrier surface [205,206]. From this perspective, exosome-coated LBNs and cell membrane-coated LBNs can be interpreted as biomembrane-based extensions of traditional LbL-LBN interface programming.

In addition, recent studies have reported hybridization strategies in which nanoparticles are wrapped with exosomes, thereby acquiring exosome-derived functionality [207,208]. Zhao et al. synthesized folic acid-SEVs@CMG by loading a carbon-dot-based nanozyme (CMG) into semen-derived extracellular vesicles (SEVs) while preserving the intact membrane function of SEVs and inserting folic acid onto the surface [209]. They exploited the principle that epidermal growth factor (EGF) present on the outer membrane of SEVs can activate epidermal growth factor receptor (EGFR) in ocular epithelial cells and reversibly open tight junctions through the EGFR–Src–myosin light chain 2 kinase–myosin light chain 2 pathway. The resulting folic acid-SEVs@CMG achieved a noninvasive delivery platform capable of reaching the posterior eye segment through topical instillation alone and demonstrated effective retinoblastoma cell killing through the enzymatic activity of the nanozyme. This study raises the possibility that replacing the carbon-dot component with an LBN possessing a solid core, such as an NLC, could enable the fabrication of an exosome-layer hybrid LbL-LBN with improved biocompatibility. It also presents an advanced strategy for exploiting the natural penetration mechanisms of exosomes without disrupting them, suggesting strong potential for future LBN-based drug delivery systems.

The exosome–LBN hybrid illustrates this extension logic most intuitively. Sato et al. prepared hybrid exosomes by fusing exosome membranes and liposome membranes using a freeze–thaw method and showed that exosome–cell interactions and cellular uptake could be altered by controlling the lipid composition of the liposome [151]. The significance of this case is not simply that exosomes and liposomes were mixed. Exosomes provide a biological interface composed of cell-derived membrane proteins, lipid composition, and tetraspanin markers, whereas liposomes provide formulation flexibility, including compositional control, introduction of external lipids, and modulation of sur-face properties. Thus, exosome–liposome hybrids can be viewed as chimeric lipid vesicles that integrate the cell-recognition function of natural membranes with the designability of synthetic lipid vesicles within a single interface.

Cell membrane-coated LBNs are important in the same context. Red blood cell mem-branes, platelet membranes, cancer cell membranes, macrophage membranes, and stem cell-derived membranes can each provide distinct biological identities [206]. For example, red blood cell membranes can be used for immune evasion and prolonged circulation, platelet membranes for vascular injury- or tumor-associated interactions, cancer cell membranes for homotypic targeting, and macrophage membranes for recognition of inflammatory tissues [210,211,212,213]. Such membrane coating differs from single-ligand conjugation. Rather than artificially introducing a single receptor–ligand pair, it reconstructs on the carrier surface complex bio-interfacial information composed of membrane proteins, lipid rafts, glycan patterns, and adhesion molecules inherently present in natural membranes [188,196]. Therefore, cell membrane-coated LBNs may enable multiple biointerfacial functions more effectively than conventional ligand-modified LBNs.

A related example is the development of leukosomes by Molinaro et al. These authors designed leukosomes by extracting membrane components, including proteins, from leukocyte membranes and integrating them onto the surface of LBNs to evade the mononuclear phagocyte system and promote transport across the endothelial vascular wall [214]. Rather than inserting a single specific ligand into the liposome surface, they incorporated adhesion-related membrane proteins present in leukocyte membranes into the lipid bi-layer to induce biomimetic interactions with inflamed vascular endothelial cells. Leukosomes maintained particle size, surface charge, drug loading, and release characteristics comparable to those of conventional liposomes, while selectively adhering to inflamed vasculature through leukocyte-derived membrane proteins. In particular, dexamethasone-loaded leukosomes selectively delivered the drug to inflamed tissue, reducing local inflammatory responses and neutrophil infiltration. This case demonstrates that cell membrane-derived biological interfaces can improve in vivo targeting and therapeutic efficiency of LBNs. In a subsequent study, the same group synthesized leukosomes as bio-mimetic nanovesicles incorporating the inflammatory targeting ability of leukocytes [215]. These vesicles were proteolipid structures reconstructed by incorporating leukocyte-derived membrane proteins into a conventional liposomal lipid bilayer, and doxorubicin (DOX) was stably encapsulated within leukosomes by remote loading. Leuko-some-DOX maintained a uniform size of approximately 120 nm and exhibited slower drug release than liposomes, likely owing to its densely organized lipid-membrane structure. In breast cancer and melanoma models, leukosomes accumulated much more strongly in tumor vasculature than conventional liposomes and showed superior tumor-growth inhibition and survival benefit compared with free DOX, supporting their potential as next-generation cancer-targeted therapeutic platforms.

These biomimetic hybrid interfaces are particularly meaningful in combination therapy systems. Simply co-loading different cargoes is often insufficient to simultaneously satisfy in vivo stability, target-tissue delivery, intracellular functional expression, and immune-microenvironment modulation [53,205]. In contrast, when anticancer drugs, anti-inflammatory agents, nucleic acid cargoes, anti-gens, adjuvants, or photothermal/photodynamic active materials are positioned within the lipid core or internal compartment, while a biomembrane-derived interface is introduced externally, internal cargo design and external biointerface design can be separated and combined [205,206]. In this architecture, the internal LBN core architecture is responsible for cargo loading, protection, and release programming, whereas the external biomimetic interface mediates immune evasion, target recognition, tissue interaction, and cell-specific uptake [212,216]. In other words, the LbL design logic of LbL-LBNs extends beyond synthetic polyelectrolyte layers toward chimeric interface design that incorporates biological membrane layers.

However, these systems should not be treated as identical to conventional LbL-LBNs. Conventional LbL-LBNs are systems in which layer number, charge reversal, thickness, and release gates are controlled primarily through electrostatic interactions, hydrogen bonding, and biopolymer deposition [16]. By contrast, the function of exosome–LBN hybrids and cell membrane-coated LBNs is governed by the composition, orientation, membrane-protein preservation, lipid–protein interactions, and source-cell identity of natural membrane-derived components [53,217]. Therefore, these platforms are more accurately viewed not as direct subtypes of LbL-LBNs, but as next-generation interface-engineered LBNs that extend the concept of terminal interface programming introduced by LbL-LBNs into a biomembrane-based format.

New challenges also arise from the manufacturing and quality-control perspectives. The composition and function of exosome- or cell membrane-derived components can vary depending on the source cell type, culture conditions, cellular state, and isolation and purification methods [217,218]. In addition, after membrane coating or membrane fusion, it is necessary to confirm whether membrane-protein orientation and functionality are preserved, whether impurities or residual nucleic acids and proteins have been removed, and whether batch-to-batch variability remains within an acceptable range. Because biomembrane-derived interfaces are not merely surface layers but functional layers carrying biological identity, conventional characterization based on size/PDI, zeta potential, and morphology is insufficient. Accordingly, additional quality attributes are required, including Western blotting, proteomics, lipidomics, membrane-marker retention, immune response, complement activation, and long-term stability [217,218,219,220].

Taken together, biomimetic hybrid interfaces represent an important direction for ex-tending the design logic of LbL-LBNs toward combination therapy and next-generation nanocarrier development. If LbL architecture provides spatial freedom for cargo positioning and release regulation, exosome- or cell membrane-derived interfaces can complement in vivo recognition, immune evasion, tissue selectivity, and intercellular interaction. Therefore, chimeric platforms such as exosome–liposome hybrids, exosome–LNPs, and cell membrane-coated LBNs should be understood not as fully established final formulations, but as transitional and promising design axes through which the hierarchical sur-face architecture of LbL-LBNs expands toward natural membrane-based interface engineering.

## 5. Engineering Hurdles and Regulatory Bottlenecks for Clinical Translation

As discussed in the preceding sections, LbL-LBNs can integrate multiple functions—biomolecular shielding, targeting, biological-barrier penetration, intracellular de-livery, and stimuli-responsive release—within a single nanocolloidal structure by coupling the cargo-loading and protective capacity of lipid cores with hierarchical surface interfaces [14,15]. However, this structural multifunctionality is not necessarily an unqualified advantage during clinical translation. Once an LbL architecture is introduced, the product identity of an LBN can no longer be defined solely by basic particle attributes. The composition, sequence, surface coverage, interfacial stability, degradation behavior, and functional contribution of each layer must also be controlled. Thus, the slow clinical and commercial progression of LbL-LBNs is not simply a consequence of insufficient therapeutic potential. Rather, it reflects the substantially higher developmental burden of demonstrating structure and function simultaneously compared with conventional LBNs. From a regulatory perspective, LbL-LBNs should be regarded not as simple combinations of formulation components, but as complex nanomedicines that integrate a core–shell structure, ligand coating, multilayer surface interface, and terminal functionality [221,222]. The key question is therefore not merely whether a multilayer structure has formed, but which therapeutic function is assigned to a given layer and whether that structure–function relationship is reproducibly maintained from manufacturing through in vivo exposure. Ultimately, the translational potential of LbL-LBNs will be determined less by the sophistication of their hierarchical architecture per se than by whether that architecture can be linked to therapeutic efficacy, safety, and standardized quality attributes.

### 5.1. Translational Gap Between Clinically Validated LBN Platforms and LbL-LBNs

Within the broader DDS landscape, LBN-based products still represent a relatively limited subset of advanced complex formulations. In the field of nanomedicine and nano-DDS, however, LBNs—particularly liposomes and LNPs—are among the most clinically validated carrier classes [221,223,224]. For liposomal products, which account for the largest number of food and drug administration (FDA)-approved LBN formulations, substantial development experience has accumulated regarding critical quality attributes such as composition, particle size, drug-to-lipid ratio, encapsulation efficiency, free drug fraction, release behavior, sterility, and long-term stability. Regulatory frameworks covering chemistry, manufacturing, and controls (CMC), pharmacokinetics, bioavailability, bioequivalence, and labeling have also been established relatively clearly, particularly by the FDA and European medicines agency (EMA) [221]. In the case of RNA-LNPs, the success of patisiran and mRNA vaccines has demonstrated that LNPs can be extended as clinical platforms for nucleic acid therapeutics and vaccines [8,225,226]. Nevertheless, FDA-approved examples to date have remained concentrated largely in conventional liposomes, lipid complexes, and LNPs. Higher-order functional LBNs with hierarchical surface architectures, such as LbL-LBNs, have not yet emerged as a clearly established class of approved products [15,221,222].

Most current LbL-LBN studies remain at the stage of preclinical efficacy validation, introduction of functional coatings, or proof-of-concept demonstration in specific disease models [16,17,18]. This gap does not imply that LbL-LBNs are less promising clinically. Rather, it reflects the much higher level of structural definition and quality control required during product development. In conventional LBNs, basic particle attributes such as lipid composition and particle size can often serve as primary quality metrics. In LbL-LBNs, however, additional parameters become relevant, including layer number, layer sequence, layer thickness, surface coverage, interlayer binding, terminal charge, ligand density, and layer-specific degradation [15,222].

Accordingly, the technical bottleneck for LbL-LBNs does not lie in the inability to generate surface functionality. It lies in demonstrating that this functionality can be quantitatively defined at the product level and manufactured with batch-to-batch reproducibility [222]. From a product-development perspective, each layer should be documented not merely as an added functional material, but as a quality-relevant component with a defined role. For example, it may be necessary to demonstrate through functional comparison studies whether an inner layer suppresses cargo leakage, whether an outer layer mediates targeting or barrier penetration, and whether removal of a specific layer measurably reduces the corresponding function [227]. This approach goes beyond formulation characterization. It establishes a structure–function relationship between layer architecture and therapeutic performance. A clinically persuasive LbL-LBN is therefore unlikely to be the most complex structure containing the largest possible number of functional layers. Rather, it should be a platform with the minimum layer architecture that materially contributes to therapeutic efficacy. Future LbL-LBN design should therefore shift from maximizing functionality toward prioritizing functional necessity, measurability, reproducibility, and regulatory explainability [15].

### 5.2. Manufacturing Reproducibility, Scale-Up Challenges, and Long-Term Stability of LbL Formulations

Unlike conventional LBNs, LbL-LBNs require sequential deposition of functional layers onto a preformed nanoparticle core, introducing additional manufacturing steps and process variables during formulation development [144,228]. Depending on the assembly strategy, LbL-LBN fabrication may involve additional washing, purification, and buffer-exchange procedures to remove unbound materials and regulate the final interfacial composition [46,228]. During these processes, parameters including pH, ionic strength, polymer concentration, mixing time, layer-to-core ratio, and purification conditions can influence layer formation, cargo retention, particle size, surface charge, and overall formulation stability [144,229]. Consequently, establishing universally applicable manufacturing conditions across different LbL-LBN formulations remains challenging, as the optimal assembly parameters are often highly dependent on the physicochemical properties of the core cargo and layer composition.

Even when similar manufacturing platforms or equipment are employed, variations in material characteristics and formulation composition may require independent optimization of solution conditions, mixing parameters, and layer-to-core ratios for each LbL-LBN system [46,144,229]. Therefore, previously established process conditions cannot always be directly transferred to newly developed formulations without additional validation. Furthermore, similar to other nanomedicines, LbL-LBNs are highly sensitive to manufacturing processes and scale-dependent variations. A formulation that demonstrates reproducible characteristics at laboratory scale does not necessarily guarantee preservation of critical quality attributes during large-scale production. Scale-up processes may introduce unexpected challenges, including particle aggregation, reduced layer integrity or layer detachment, cargo loss, decreased production yield, and increased batch-to-batch variability. Accordingly, stepwise process control and scale-dependent comparability assessments are essential to ensure consistent structural and functional properties during manufacturing expansion.

Such formulation-specific optimization and repeated validation requirements increase the overall process burden and may ultimately limit the industrial scalability of LbL-LBN platforms [46,229,230]. To facilitate future translation, manufacturing strategies that integrate microfluidic-based core nanoparticle production with controlled and continuous or semi-continuous layer deposition should be considered. In parallel, advanced process monitoring approaches are required to verify whether the structural integrity of external layers and their associated cargo-protection functions are maintained throughout complex manufacturing workflows and scale transitions [229,230].

Long-term stability represents another critical challenge for the development and quality assessment of LbL-LBN products. In addition to conventional stability concerns associated with LBNs, including particle aggregation, lipid oxidation, cargo leakage, and changes in particle size, LbL-LBNs require evaluation of the structural and functional stability of their multilayer architectures [142,229,230]. Unlike passive surface coatings, each layer within an LbL-LBN can contribute distinct functions, including surface-charge regulation, targeting, cargo protection, and controlled release [103,228,231,232]. Therefore, layer detachment or reorganization during storage may alter not only the physical properties of the nanoparticles but also the accessibility and presentation of terminal ligands, exposure of underlying layers, and retention or release behavior of layer-associated cargos, even without substantial changes in particle size [103,142,230,231].

This issue becomes particularly important for systems in which nucleic acids, such as siRNA, or functional biomolecules, such as peptides, are incorporated as components of the multilayer architecture. Loss of multilayer integrity may alter the degree of cargo protection, molecular accessibility, or release timing, potentially resulting in premature exposure, uncontrolled release, or reduced therapeutic activity [103,232]. Such structural alterations can ultimately reshape the biological identity of the nanoparticle surface and modify interactions with target cells and biological barriers. Therefore, stability evaluation of LbL-LBNs should extend beyond conventional physicochemical stability measurements and include whether each layer maintains its intended structural role and biological function throughout storage and handling conditions [103,229,231,232].

### 5.3. Critical Quality Attributes, Analytical Standardization, Stability Evaluation, and Regulatory Considerations

Under the regulatory framework established by the U.S. Food and Drug Administration (FDA), conventional drug development follows a sequential pathway in which preclinical evaluation is followed by the submission of an Investigational New Drug (IND) application, phase I clinical trials for safety and dose determination, phase II trials for efficacy and adverse-event evaluation, and phase III trials in larger patient populations to further validate therapeutic benefit and safety, ultimately leading to regulatory review through a New Drug Application (NDA) or Biologics License Application (BLA) [233,234,235,236]. Throughout this process, regulatory assessment requires not only clinical safety and efficacy data but also comprehensive quality information regarding formulation composition, manufacturing processes, stability, and production control [103,234,235,236]. Additional studies and data supplementation may be required when new safety concerns or quality variations are identified during development, while late-stage clinical trials further evaluate adverse events that may not be sufficiently captured in earlier studies due to the larger patient population involved [233,235]. These requirements can become even more demanding for nanomedicines with complex structures and dynamic in vivo behaviors. The FDA has emphasized that, for certain nanomedicines, physicochemical properties may influence biodistribution, residence time, and interactions with biological components, potentially requiring additional physicochemical, nonclinical, and clinical characterization [222]. Therefore, successful clinical translation of LbL-LBNs requires analytical strategies capable of correlating structural composition, cargo stability, and biological function within a unified evaluation framework [222,237].

Quality assessment of LbL-LBNs should not be limited to reporting individual physicochemical parameters, such as particle size, surface charge, morphology, and composition, but should instead define how each parameter reflects specific critical quality attributes (CQAs) and contributes to the overall structure–function relationship of the formulation [230,231,237]. In addition to evaluating formulation stability and release characteristics through cargo loading efficiency and release behavior, systems incorporating nucleic acid cargos, such as siRNA, require assessment of their functional outcomes, including the resulting gene regulation activity [231,237]. Furthermore, safety evaluation should consider not only the intrinsic toxicity of individual layer components but also the biological responses arising from the assembled multilayer architecture and its resulting surface properties. For hybrid interfaces incorporating biomaterial-derived membranes, additional quality attributes may include source-material variability, preservation of membrane protein functionality, residual impurities, and potential immunogenicity [222,231,238].

Although comprehensive physicochemical, functional, and safety evaluations provide multidimensional characterization of LbL-LBN quality, individual in vitro analyses alone remain insufficient to fully predict whether the intended structure and function are preserved under physiological conditions [237,239]. Following administration, nanoparticles undergo complex interactions involving protein corona formation through plasma protein adsorption, recognition and clearance by the mononuclear phagocyte system, enzymatic degradation, interactions with cellular and tissue barriers, and tissue-specific distribution, all of which can alter nanoparticle identity and therapeutic exposure profiles [222,230]. Such biological influences have also been observed in LbL-based systems, where multilayer organization and terminal-layer composition can affect in vivo stability, nonspecific cellular uptake, and biodistribution. Moreover, nanoparticle mechanical properties have been reported to influence elimination half-life and accumulation patterns in major organs and tumor tissues [240]. Therefore, evaluation of LbL-LBNs should move beyond independent interpretation of individual physicochemical measurements and instead establish how multilayer integrity and layer-specific functions collectively influence cargo delivery, pharmacokinetics, and in vivo distribution [210,222,231,237,240].

The relationship between LbL-LBN structure, biological function, and in vivo behavior is also directly connected to long-term safety evaluation [210,222,240]. Unlike conventional small-molecule drugs, nanomedicines can alter not only the tissue distribution and half-life of the therapeutic cargo but also introduce additional considerations related to the biodistribution, persistence, and clearance of the carrier itself [237]. The FDA has highlighted that non-degradable nanomaterials may exhibit prolonged retention or accumulation in the body, potentially resulting in chronic exposure-related effects, and therefore recommends evaluation of biodistribution, accumulation, and clearance according to material-specific characteristics [221]. Accordingly, LbL-LBN development should extend beyond short-term efficacy and toxicity assessment and evaluate the persistence and clearance of both core and layer components, off-target tissue distribution, and safety profiles following repeated administration in accordance with formulation characteristics [210,221,240]. These additional evaluation requirements represent important developmental considerations that may influence the translational pathway of LbL-LBN platforms [46,221,241].

The clinical translation of LbL-LBNs ultimately requires integration of accumulated evidence from formulation design, manufacturing optimization, physicochemical characterization, stability evaluation, biological function assessment, and safety validation into a coherent quality and regulatory framework [221,237,241]. Although LbL-LBNs provide substantial design flexibility in cargo organization, release regulation, targeting, and combination therapy, their clinical and commercial potential can only be realized when these advantages are achieved through reproducible manufacturing, long-term structural stability, and standardized evaluation strategies [103,210,241]. Therefore, future development of LbL-LBNs should prioritize the establishment of rational multilayer architectures that preserve essential therapeutic functions while minimizing unnecessary structural complexity. In particular, evaluation strategies should be developed to systematically connect multilayer structure, functional performance, and in vivo behavior, enabling changes in layer integrity and functionality to be directly correlated with biodistribution, cargo delivery efficiency, and long-term safety outcomes [46,221,241]. Ultimately, successful clinical translation of LbL-LBNs will require standardized analytical frameworks that move beyond individual physicochemical characterization and provide an integrated understanding of how each layer contributes to formulation stability, biological function, and therapeutic performance [221,231,240,241].

## 6. Conclusion and Future Perspectives

Through major advances in core architecture—from lipid emulsions to ionizable LNPs—LBNs have firmly established their clinical potential and commercial impact in the delivery of poorly soluble small-molecule drugs, gene therapeutics, RNA vaccines, and immunomodulatory agents. However, as the therapeutic targets pursued in modern nanomedicine become more biologically specific and as pathological microenvironments become increasingly complex, core-centered optimization or one-dimensional modification based on single mixed interfaces is encountering clear limitations. Attempts to simultaneously coordinate conflicting functional requirements on a single interface—colloidal stability, serum-protein shielding, disease-target recognition, suppression of premature leakage, and stimuli-responsive activation—inevitably generate functional non-orthogonality.

LbL-based interface engineering has emerged as one of the most compelling hierarchical design frameworks for addressing this structural dilemma. LbL technology is not a process that simply adds a passive protective coating onto the LBN surface. Rather, it is an active form of interface programming that reconstructs the colloidal surface as a three-dimensional, multicompartmental infrastructure by sequentially depositing functional layers around the lipid core using thermodynamic templates such as complementary charge and hydrogen bonding. The preclinical design examples discussed in this re-view illustrate the multifunctional potential of LbL architecture. More recently, this concept has evolved beyond simple polymeric layering toward biomimetic hybrid approach-es in which natural lipid bilayers derived from exosomes, red blood cells, or tumor cells are transplanted onto the interface. In this broader context, LbL-based interface engineering is becoming a central design principle for distributing functional modules across complex disease microenvironments in a tissue- and context-dependent manner.

For the distinctive potential of LbL-LBNs to move beyond preclinical achievement and translate into clinically useful therapeutics, however, the critical weaknesses introduced by structural sophistication must be addressed directly. As multilayer structures become increasingly complex in the name of multifunctionality, manufacturing yield, batch-level quality control, and regulatory acceptance become substantially more difficult. This complexity increases the burden of controlling layer sequence, surface coverage, interlayer stability, outermost-layer identity, ligand accessibility, storage stability, and bio-logical potency. Accordingly, an orthogonal characterization framework that combines optical, physicochemical, and surface-chemical analytical methods should be established to quantitatively define the therapeutic contribution and stability of each layer. From an industrial perspective, it is essential to move beyond laboratory-scale multistep adsorption processes that rely on repeated centrifugation and washing. Microfluidic and closed continuous-assembly platforms offer a practical route forward by enabling precise control of fluid mixing under nanoscale laminar-flow conditions, reducing variability in layer coverage, and supporting GMP-compatible aseptic production. When these process technologies are incorporated, QbD principles should be introduced from the earliest stages of development. The physicochemical variables of each layer should be defined as CQAs in relation to structural morphology and biological potency, and comparability should be rigorously supported by data.

Taken together, future LbL-LBN development should avoid functional excess—the uncritical addition of numerous novel or rare polymer layers simply to increase apparent sophistication. LbL architecture provides a powerful framework for reconstructing the surface architecture of LBNs when single-layer or mixed-interface designs are insufficient to achieve orthogonal control over delivery functions. The field should now move toward selecting a minimum functional architecture that is essential for the biological-barrier penetration and release kinetics required by a given target disease. Interfaces should be built primarily from materials with strong biocompatibility profiles and accumulated regulatory experience, thereby maximizing process reproducibility and translational feasibility. The long-term impact of LbL-LBNs will not come from pursuing ever more complex multilayer systems. It will come from systematically converting hierarchical interface design into simplified, measurable, and clinically deployable products. From this perspective, LbL-LBNs should be regarded both as a next-generation interface-engineering strategy for precision delivery and as a conceptual bridge toward broader biomimetic and hybrid nanocarrier design.

## Figures and Tables

**Table 1:** Representative LBN platforms, core architectures, and remaining formulation limitations.

Platform	Core Architecture	Main Cargo	Remaining Limitation	Ref.
Lipid emulsion	Liquid lipid droplets stabilized by phospholipids, surfactants, or co-surfactants	Hydrophobic drugs, lipophilic nutrients, anesthetics, poorly water-soluble small molecules	Physical instability, coalescence/Ostwald ripening, rapid drug release, limited cargo compartmentalization, weak surface programmability	[24]
Cubosome	Bicontinuous lyotropic cubic phase formed by amphiphilic lipids, with internal aqueous nanochannels	Hydrophobic, amphiphilic, and some hydrophilic drugs; peptides and bioactives	Phase transition, high viscosity, stabilizer dependence, manufacturing complexity, limited clinical translation, difficult surface/interface control	[6]
Liposome	Phospholipid bilayer with aqueous core	Hydrophilic drugs, hydrophobic drugs, peptides, proteins	Drug leakage, vesicle fusion, limited hydrophobic drug loading, serum protein-induced destabilization	[25,26]
SLN	Solid lipid matrix	Hydrophobic drugs, chemically labile small molecules	Drug expulsion caused by lipid crystallization, limited loading capacity	[27]
NLC	Solid lipid and liquid lipids	Hydrophobic drugs, multi-drug combinations	Possible lipid polymorphic transition, surfactant-related toxicity, uncontrolled surface identity	[27,28]
LNP	Ionizable lipid-based nucleic acid with helper lipids and PEG-lipid	siRNA, mRNA, miRNA, gene-editing cargo	Immune stimulation, PEG-related shielding, extrahepatic targeting limitation	[29,30]

**Table 2:** Interfacial bottlenecks of conventional LBN surface modification strategies and their implications for LbL-based hierarchical design.

Conventional Modification	Interface Function	Interfacial Bottleneck	LbL Design Rationale	Ref.
Hydrophilic shielding	Hydration/steric stabilization, reduced protein adsorption, prolonged circulation	Ligand shielding, reduced cellular accessibility	Separate shielding layer from targeting layer	[34,35]
Charge modulation	Electrostatic stabilization, membrane interaction, nucleic acid binding	Cytotoxicity, protein adsorption, nonspecific uptake	Decouple charge layer from terminal biointerface	[44,45]
Ligand conjugation	Receptor recognition and active targeting	Ligand orientation, density, accessibility, corona masking	Present ligand as terminal layer	[36]
Polymer/biopolymer coating	Core protection, leakage suppression, mucoadhesion, release delay	Coupled thickness, charge, permeability, degradation	Use independent protective or release-control layer	[37,38]
Stimuli-responsive modification	Triggered release, de-shielding, environmental activation	Stability responsiveness trade-off	Place responsive layer as intermediate functional layer	[41,42]

**Table 3:** Modern alternative surface-engineering strategies and their structural relationship to LbL design.

Strategy	Core Mechanism	Advantages	Structural Relationship to LbL	Ref.
		Limitations		
Click chemistry-based conjugation	Bioorthogonal covalent coupling of ligands onto a pre-formed nanoparticle surface	High chemoselectivity under mild aqueous conditions	Can be adopted as the conjugation chemistry within an LbL terminal layer, rather than as a substitute for layer-level functional separation	[47,48]
		requires pre-installed chemical handles and remains confined to a single surface layer		
Post-insertion ligand attachment	Insertion of pre-formed lipid–PEG–ligand micelles into a preassembled lipid bilayer	Avoids ligand exposure to harsh formulation conditions	Structurally analogous to terminal-layer deposition in LbL, but lacks sequential separation of shielding and targeting into distinct layers	[49]
		Offers limited control over ligand density/orientation and keeps shielding and targeting coupled in one leaflet		
Membrane fusion strategies	Direct fusion of liposomal, exosomal, or viral-derived membranes with target cell or carrier membranes	Enables direct cytoplasmic delivery bypassing endosomal entrapment	Fusogenic lipids/peptides can be incorporated as one discrete intermediate layer within an LbL architecture rather than as a standalone mechanism	[50]
		Fusion efficiency is highly cell-type dependent and difficult to control in vivo		
Polymer–lipid hybrid nanoparticles	Single-step co-assembly of polymer and lipid components into one blended core–shell structure	Combines polymeric stability with lipid biocompatibility in one simplified step	LbL differs by depositing each function as a discrete, sequential layer rather than pre-blending components into one hybrid shell	[51]
		Functions remain blended within a single shell rather than spatially partitioned		
Biomimetic cell membrane coating	Extrusion- or sonication-based coating of natural cell-derived membranes onto a synthetic nanoparticle core	Confers innate immune evasion and homologous targeting	Membrane fragments could conceptually serve as one terminal LbL layer, but coating alone lacks layer-level control over composition and thickness	[52,53]
		Shows batch-to-batch variability and frequently incomplete coating coverage		
DNA origami-based surface engineering	Programmable self-assembly of DNA strands into addressable nanoscale scaffolds	Sub-nanometer spatial precision in ligand placement	Offers superior single-ligand precision but lacks LbL’s capacity for bulk multilayer functional stacking	[54]
		High cost, nuclease sensitivity, and limited scalability		

**Table 4:** Layer materials and functional interface modules in LbL-LBNs.

Interface Module	Typical Layer Position	Representative Materials	Main Role in LbL-LBN	Ref.
Cationic anchoring/cargo-binding layer	Inner layer	Chitosan, protamine, poly-L-arginine, poly-L-lysine, Polyetherimide	Electrostatic anchoring, charge reversal, cationic priming of lipid surface	[95,96]
	Intermediate layer	Chitosan, protamine, poly-L-arginine, poly-L-lysine, Polyetherimide	Nucleic acid binding, interfacial cargo layer formation, bridge to anionic layer	[95,103]
	Terminal layer	Chitosan, protamine, poly-L-arginine, poly-L-lysine,	Membrane interaction, mucoadhesion, uptake enhancement	[44,96]
Anionic shielding/release-modulating layer	Intermediate layer	Alginate, HA, dextran sulfate, heparin, TPP, PP, polyphosphates	Charge compensation, shielding, release delay, de-shielding gate	[39,97,98]
	Terminal layer	HA, alginate, dextran sulfate, heparin, polyphosphates	Hydrated anionic surface, biocompatibility, surface charge masking, receptor-accessible HA presentation	[39,97,98]
Protective/stealth layer	Terminal layer	PEG, zwitterionic polymer, neutral hydrophilic polymer, hydrated polysaccharide, HA	Terminal hydration layer, anti-fouling surface, biological identity modulation	[34,35,99]
	Outer shielding layer	PEG, zwitterionic polymer, neutral hydrophilic polymer, hydrated polysaccharide	Protein adsorption reduction, colloidal stability, prolonged circulation, nonspecific interaction suppression	[99,104]
Targeting/biointerface layer	Terminal layer	HA, folate, peptide, antibody fragment, aptamer, transferrin	Receptor recognition, biological identity control, cell-selective binding	[36,39,100]
Stimuli-responsive layer	Intermediate layer	pH-, redox-, enzyme-, ROS-responsive polymers or linkages; TPP, PP, polyphosphates	Triggered exposure, charge transition, de-shielding, release gating	[41,42,101]
	Terminal or outer-responsive layer	pH-sensitive PEG, enzyme-cleavable polysaccharide, redox-cleavable polymer, ROS-responsive polymer	Conditional shielding, environment-dependent activation, terminal layer removal or loosening	[60,105,106]
Nucleic acid-based functional layer	Interfacial layer	siRNA, miRNA, DNA, CpG oligonucleotide	Gene regulation, immunostimulation, surface-associated cargo layer formation	[17,18,102]
	Intermediate layer	siRNA, miRNA, DNA, CpG oligonucleotide	Interlayer cargo depot, electrostatic complexation with cationic layer, decomplexation-mediated release	[102,103]

**Table 5:** Representative in vitro quantitative outcomes of LbL surface engineering.

Metric	System	Quantitative Outcome	Ref.
Particle size shift	Chitosan/alginate layer-by-layer system	152.1 nm → 194.6 nm → 228.9nm	[71]
Polydispersity index (PDI)		0.25 → 0.31 → 0.36	
Zeta potential/charge reversal		−3.8 mV → +8.4 mV → −27.8 mV	
Intracellular cargo dissociation timing	Doxorubicin loaded liposome coated with poly-L-arginine (PLA), siRNA, PLA, and hyaluronic acid for triple-negative breast cancer	siRNA–liposomal core co-localization at 30 min → dissociation by 240 min	[103]
Layer-position-independent gene silencing	Five-layered hyaluronic acid–lipid nanoparticle, with luciferase-targeting siRNA positioned in the 1st vs. 3rd layer	Comparable gene-silencing efficiency regardless of layer position	[17]
Enzyme-responsive cellular uptake	Alkaline phosphatase (ALP)-responsive protamine-coated nanostructured lipid carrier, shielded with sodium tripolyphosphate or sodium polyphosphate	Increased uptake upon ALP-mediated de-shielding; reduced uptake under phosphatase inhibitor cocktail treatment	[74]
Enzyme-triggered colonic release (in vitro)	Budesonide-loaded solid lipid nanoparticle with two-layer polyelectrolyte coating	Minimal release in simulated gastric fluid and simulated intestinal fluid vs. markedly increased release in simulated colonic fluid	[117]
pH-triggered release (in vitro)	Spectinomycin-loaded layer-by-layer hybrid nanoparticles	Higher cumulative release at pH 6.0 vs. pH 7.4	[119]
Sustained release comparison (in vitro)	Celastrol-loaded layer-by-layer liposome coated with pectin/trimethylated chitosan vs. free Cel and Cel-loaded liposome	Slowest release profile among the three formulations, evaluated over simulated gastric fluid (2 h), simulated intestinal fluid (4 h), and simulated colonic fluid (90 h)	[120]

## Data Availability

No new data were created or analyzed in this study. Data sharing is not applicable to this article.
